# Chemical Ontogeny in Plants: Developmental Dynamics of Specialized Metabolism and Implications for Ecology, Quality Control, and Bioprospecting

**DOI:** 10.3390/plants15182756

**Published:** 2026-09-09

**Authors:** Dannily Augusto Rebouças, Lorraynne Oliveira-Souza, Tamara do Nascimento da Silva, Rodrigo Guimarães de Deus, Orlando Maia Barboza, Clara Cabral Noronha, Philippe Barreto de Almeida, Gustavo Borges Andrade, Davyson de Lima Moreira, Ygor Jessé Ramos

**Affiliations:** 1 Farmácia da Terra Laboratory, Faculty of Pharmacy, Federal University of Bahia, Rua Barão de Jeremoabo, 147, Ondina, Salvador 40170-115, BA, Brazil; dannily_rs@hotmail.com (D.A.R.); lorraynneoliveira@jbrj.gov.br (L.O.-S.); tmarasilva@gmail.com (T.d.N.d.S.); deus.r.g.1@gmail.com (R.G.d.D.); orlandomaiabarboza@gmail.com (O.M.B.); claracnoronha@gmail.com (C.C.N.); philippebarretto@gmail.com (P.B.d.A.); andrade.gustavo@ufba.br (G.B.A.); 2 Research Institute of the Rio de Janeiro Botanical Garden, Rua Pacheco Leão 915, Jardim Botânico, Rio de Janeiro 22460-030, RJ, Brazil

**Keywords:** chemical ontogeny, ontogenetic variation, plant development, specialized metabolism, metabolomics, chemical ecology, pharmacognosy, quality control

## Abstract

Plant specialized metabolism changes throughout development, but developmental context remains inconsistently incorporated into phytochemical interpretation. This integrative narrative review synthesizes evidence on chemical ontogeny in plants, defined here as the qualitative and quantitative reorganization of specialized metabolism along plant and organ development. We distinguish ontogenetic variation from phenology, seasonality, organ maturation, developmental plasticity, and environmentally induced chemical plasticity, and examine the genetic, hormonal, anatomical, ecological, and analytical processes that generate stage-dependent chemical profiles. Across seeds, seedlings, vegetative organs, reproductive structures, fruits, and senescent tissues, the evidence shows recurrent changes in metabolite abundance, composition, spatial allocation, and bioactivity. These trajectories can alter ecological interactions, marker stability, harvest recommendations, and the reproducibility of botanical raw materials. Analytical strategies should therefore be selected according to the biological question and available resources, ranging from validated targeted or single-omics designs to spatial, single-cell, and multi-omics integration when mechanistically justified. Chemical ontogeny is proposed not as evidence that developmental metabolism is newly discovered, but as an operational framework for stage-aware sampling, reporting, interpretation, quality control, and bioprospecting.

## 1. Introduction

Plant chemical composition is developmentally contingent rather than fixed. At any sampling point, the observed chemical phenotype reflects the developmental state of the individual and sampled organ, together with genotype, tissue identity, environment, and recent ecological history. Plant development itself is spatially and temporally regulated through cell division, differentiation, signaling, and organ formation [1,2,3]; specialized metabolism is embedded within these processes rather than superimposed on them.

Developmental variation in plant chemistry is not a new observation. It has long been studied in chemical ecology, pharmacognosy, developmental physiology, and metabolomics. The unresolved problem is methodological and interpretative: developmental effects are often reported under partially overlapping labels such as seasonal, phenological, age-related, organ-specific, or environmentally induced variation [2,4]. Here, chemical ontogeny is used as an organizing framework for asking which part of chemical variation is associated with developmental progression, at which biological scale, and with what consequences for inference.

Specialized metabolites comprise broad chemical classes, such as terpenoids, phenolic compounds, sulfur-containing compounds, alkaloids, glucosinolates, and other nitrogen-containing derivatives. Although these compounds were historically interpreted as accessory products of metabolism, chemical ecology has demonstrated that they play central roles in defense, ecological communication, stress adaptation, and the mediation of biotic interactions [5,6,7,8]. More recently, this view has been expanded by the recognition that many of these metabolites also participate in growth regulation, cell differentiation, endogenous signaling, and the integration between development and defense, thereby reducing the rigid separation between primary, secondary, and hormonal metabolism [9,10,11]. Therefore, specialized metabolism should be understood as an active component of the plant’s functional organization, rather than merely as a chemical repertoire directed toward external interactions.

In this context, ontogeny constitutes a central variable for understanding plant chemical biology. Organs and tissues at different developmental stages exhibit distinct metabolic priorities, potential differences in medicinal value, ecological vulnerabilities, and adaptive values. Optimal defense theory and studies on the ontogeny of plant defense indicate that the allocation of defensive metabolites varies according to the functional value of tissues, the risk of attack, the cost of replacement, and their contribution to reproductive performance [12,13,14,15,16]. Experimental and meta-analytical evidence reinforces that chemical defense changes throughout development, although the patterns vary according to the plant group, metabolite type, and ecological interaction considered [14,15].

Despite its importance, classical phytochemistry has often relied on samples collected at a single time point, from one organ, or without precise developmental characterization [4]. Although this approach has supported the discovery of natural products and chemical markers, it may overlook variation associated with plant age, ontogenetic stage, organ position, physiological status, and senescence, thereby limiting comparisons and ecological, pharmacognostic, chemotaxonomic, and chemophenetic interpretations [2,4]. Developmentally resolved approaches help overcome this limitation by treating specialized metabolites as dynamic chemical trajectories organized in time and space. Advances in metabolomics, chromatography, mass spectrometry, chemical imaging, and multi-omics have improved the mapping of metabolites across organs, tissues, and cell types and have strengthened links between chemical variation, biosynthetic pathways, regulatory mechanisms, tissue differentiation, and compartmentalization [11,17,18].

Empirical studies support this developmental perspective. In *Piper*, for example, ontogenetic stages show distinct qualitative and quantitative chemical profiles with ecological, taxonomic, and pharmacognostic relevance [19]. Such findings are consistent with chemophenetic and chemodiversity approaches that interpret plant chemistry as a dynamic, multiscale property [4]. This framework has practical implications for ecology, quality control, traceability, pharmacognosy, and bioprospecting because developmental stage may influence chemical signals, marker stability, bioactivity, authenticity, and the occurrence of rare or transient metabolites. Accordingly, metabolomic, chemophenetic, and integrative approaches can improve the interpretation of plant chemical diversity beyond models based on isolated markers [4,20,21].

Chemical ontogeny also provides a relevant basis for protocols related to cultivation, collection, processing, and standardization. The definition of the optimal harvest time should not depend solely on available biomass or apparent phenological phase, but rather on the relationship among plant development, the accumulation of metabolites of interest, chemical stability, intraspecific variability, and therapeutic or biotechnological purpose. In medicinal plants, this perspective is particularly important because the ontogenetic stage may directly affect chemical composition, marker concentration, the occurrence of bioactive metabolites, and the consistency of analytical profiles used in quality control. Thus, understanding the ontogenetic dynamics of specialized metabolism may contribute to greater traceability, reproducibility, and quality of plant raw materials in productive chains involving medicinal, aromatic, food, and pharmaceutically relevant plants [4,19].

This review therefore does not claim novelty for the established observation that metabolism changes with development. Its added value is to convert that premise into a stage-aware framework for phytochemical research. Specifically, we distinguish ontogenetic variation from phenology, seasonality, organ maturation, and environmental plasticity; synthesize mechanistic evidence from molecular regulation to tissue differentiation; organize recurrent chemical trajectories across the plant life cycle; evaluate analytical strategies at different levels of technical complexity; and derive operational recommendations for sampling, reporting, quality control, and bioprospecting. The aim is to improve causal interpretation and comparability rather than to replace established developmental, ecological, metabolomic, or chemophenetic frameworks.

## 2. Critical Synthesis and Discussion

### 2.1. Conceptual Foundations of Chemical Ontogeny

The conceptual formulation of chemical ontogeny initially requires the epistemological delimitation of the term *ontogeny*. The word derives from the articulation of the Greek roots *ón* and *óntos*, related to being, the entity, or that which exists, and *génesis*, associated with origin, formation, generation, and development. In its modern biological sense, the term was introduced and systematized in the nineteenth century by Ernst Haeckel, especially in Generelle Morphologie der Organismen, published in 1866, in the context in which the author distinguished ontogeny from phylogeny and formulated the later-criticized proposition that ontogeny recapitulates phylogeny [22,23,24].

Although Haeckel was situated within the field of evolutionary zoology, the notion of ontogeny should not be understood as a category restricted to animal embryogenesis. Since its modern formulation, the concept has had a broader scope, referring to the process through which an organism is formed, differentiates, and transforms over time. In this sense, ontogeny designates the individual history of development, encompassing the origin of the organism and its successive phases of growth, maturation, reproduction, and senescence.

In plant biology, this notion has acquired its own specificities. Unlike animals, plants exhibit modular development, indeterminate growth, continuous meristem differentiation, and the successive formation of organs throughout life. Thus, plant ontogeny is not restricted to an initial embryonic sequence, but involves the reiterated production of leaves, branches, flowers, fruits, lateral roots, storage tissues, secretory structures, and architectural units. This condition makes the plant a particularly suitable organism for ontogenetic analysis, since its form, physiology, and chemical composition are continuously reorganized over time.

The incorporation of ontogeny into plant biology occurred through morphology, organography, anatomy, physiology, and, subsequently, developmental biology. The morphological tradition represented by Goethe, Hofmeister, Goebel, and Troll provided a historical basis for understanding the plant as an organism constituted by the successive transformations of organs, tissues, and architectural patterns [25,26,27,28,29,30,31,32,33,34]. Thus, the application of ontogeny to plant biology arose from the need to understand the plant as an organism whose identity is progressively constructed through temporal, reiterative, and multiscale processes.

**Figure 1:**
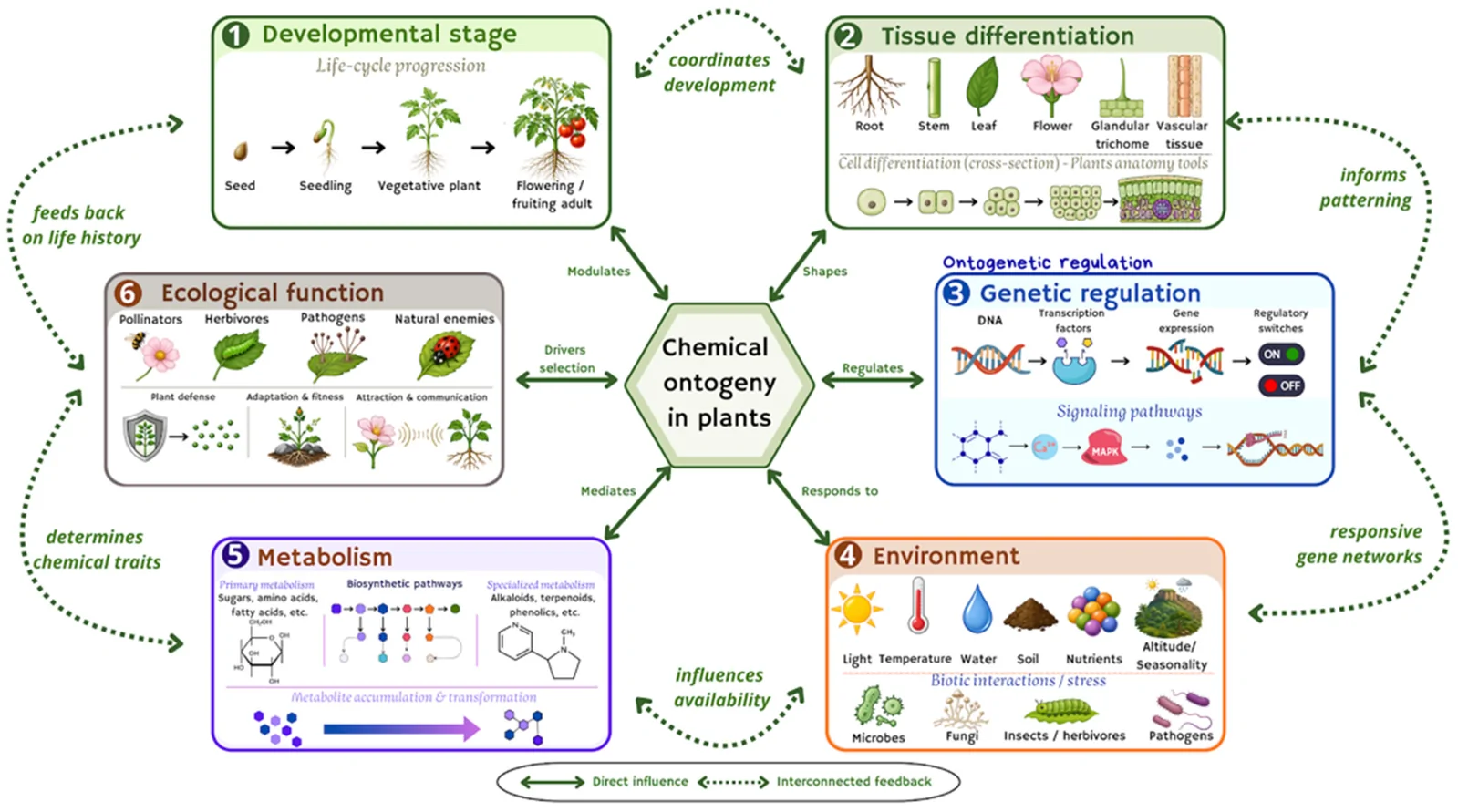
Conceptual framework of chemical ontogeny in plants.

This point is essential for the formulation of chemical ontogeny. If organs, tissues, and secretory structures are formed at distinct times and under different physiological states, plant chemical composition must also be interpreted as the result of a developmental trajectory. These interdependent dimensions are synthesized in Figure 1, which proposes chemical ontogeny as an integrative framework linking developmental stage, anatomical differentiation, genetic regulation, environment, metabolic pathways, and ecological function. Young leaves, fully expanded leaves, floral buds, flowers at anthesis, immature fruits, mature fruits, and senescent tissues do not merely represent anatomical or phenological categories. Each of these structures corresponds to a specific ontogenetic condition, with a given biosynthetic competence, allocation capacity, compartmentalization pattern, and ecological function.

The expression *chemical ontogeny* has its own conceptual trajectory. Before its consolidation within the phytochemical field, the idea appeared in physiological and embryological studies focused on the chemical composition of developing organisms. Baldwin [35], when discussing chemical processes during the development of amphibian eggs, had already employed formulations related to the chemical dimension of ontogenesis. A few years later, Murray [36] explicitly used the expression chemical ontogeny when arguing that the chemical constitution of organisms should be analyzed across successive stages of life, correlating chemical composition, functional differentiation, growth, and development.

These records demonstrate that chemical ontogeny did not originally emerge as a term restricted to phytochemistry or pharmacognosy. Rather, it expressed a broader biological concern: understanding how the chemical constitution of an organism is progressively reorganized during the formation, differentiation, and maturation of its structures. This perspective shifts biological chemistry from a merely compositional view to a temporal view, in which substances, metabolic pathways, and chemical profiles are analyzed as a function of the individual history of the organism [35,36].

In the plant field, one of the earliest explicit records of the expression chemical ontogeny appears in the work of James B. McNair, in the article “Some comparisons of chemical ontogeny with chemical phylogeny in vascular plants”, published in Lloydia in 1945 [37]. The merit of this work lies in bringing chemical variation throughout individual development closer to the chemical variation observed at phylogenetic scales. In doing so, McNair anticipated issues that would later be revisited by chemotaxonomy, comparative phytochemistry, chemical ecology, and chemophenetics.

In pharmacognosy, Hegnauer [38] represents an important milestone by discussing the theoretical significance of chemical-ontogenetic and chemical-systematic considerations in medicinal plants. This approach shifted chemical ontogeny toward an applied concern: the content and composition of bioactive constituents are not fixed properties of the species or of the herbal drug, but vary according to the organ, tissue age, developmental stage, and time of collection. Thus, the chemical-ontogenetic perspective came to have direct implications for the authenticity, quality, standardization, marker selection, and reproducibility of botanical raw materials.

Chemical ontogeny may be defined as the metabolic dimension of plant development. It corresponds to the set of qualitative and quantitative changes in the production, allocation, transformation, transport, compartmentalization, and degradation of specialized metabolites throughout the life trajectory of the plant. The concept does not only designate the temporal variation of chemical compounds, but rather the developmental organization of the chemical phenotype in relation to specific phases, organs, tissues, cell types, and physiological states.

This definition moves beyond the interpretation of chemical composition as a fixed property of the species. From an ontogenetic perspective, the plant does not only possess an average chemical profile or a stable phytochemical signature. Rather, it expresses metabolic repertoires that are differentially organized according to developmental phase, position within the plant, physiological condition, cellular differentiation, and environmental context. The chemical phenotype should therefore be understood as a dynamic, multiscale, and historically situated property [4].

Organs and tissues at different stages of formation, maturation, reproduction, dormancy, and senescence occupy distinct positions along the plant ontogenetic trajectory. Each condition involves specific physiological demands, degrees of exposure to herbivores and pathogens, ecological functions, and biosynthetic capacities. Accordingly, chemical ontogeny provides a conceptual basis for integrating plant development, specialized metabolism, chemical ecology, pharmacognosy, quality control, and bioprospecting [19,39,40,41,42,43,44].

From a terminological standpoint, it is essential to distinguish chemical ontogeny from *ontogenetic chemical variation*. Ontogeny is the noun that designates the overall process of individual development over time. *Ontogenetic* is an adjective that qualifies events, trajectories, stages, patterns, or variations related to this process. Thus, appropriate formulations include *ontogenetic variation*, *ontogenetic change*, *ontogenetic trajectory*, *ontogenetic pattern*, and *ontogenetic stage*.

The use of ontogenetic as an autonomous noun should be avoided because it may generate grammatical and conceptual ambiguity. Similarly, *chemical ontogenetic* is appropriate only when used adjectivally, as in *chemical-ontogenetic approach* or *chemical-ontogenetic analysis*. Thus, chemical ontogeny should designate the field or analytical framework, whereas ontogenetic chemical variation should designate observable developmental changes in the metabolic profile.

It is also necessary to differentiate ontogeny from phenology. Phenology refers to the timing of recurrent life-cycle events, such as budburst, flowering, fruiting, seed dispersal, and leaf senescence, often in relation to seasonal, climatic, and ecological factors. Ontogeny, in turn, describes how the organism develops along its individual trajectory. Thus, flowering may be considered a phenological event when analyzed in relation to the calendar, photoperiod, or climatic regime, but it may also be interpreted as an ontogenetic transition when analyzed as a developmental phase change [41,42,43,45,46,47].

This differentiation is central to avoiding misinterpretations in phytochemical studies. A change in the content of a metabolite during flowering may result from the ontogenetic transition to the reproductive phase, from a specific phenological condition, from seasonal variation, or from interactions among these factors. Therefore, the analysis of chemical ontogeny requires careful description of the plant material, and whenever possible, an experimental design capable of separating age, developmental stage, environment, and seasonality.

Chemical ontogeny is based on the premise that specialized metabolism constitutes a dynamic developmental trait [7,11,48,49]. The presence of dynamic *metabolons*, that is, biosynthetic complexes organized spatially and temporally, reinforces the idea that metabolic production is coordinated in space and time, favoring efficiency, metabolic channeling, and integrated regulation of chemical pathways [50]. In this context, specialized metabolites should not be treated merely as products of biosynthetic pathways, but as active components of the functional organization of the plant.

Plant chemical defense offers a particularly clear example of this dynamic. Seedlings, young leaves, reproductive tissues, and organs of high adaptive value may show greater investment in defensive compounds, depending on the risk of herbivory, the cost of tissue replacement, and the contribution of the organ to reproductive success [15,51]. Therefore, the chemical composition of a plant is not merely a taxonomic expression of the species; it is also the transient manifestation of a metabolic network undergoing continuous development.

Chemical ontogeny should be articulated with the concepts of heteroblasty and phase transition. Heteroblasty corresponds to the expression of distinct forms throughout the development of the same plant, especially in relation to juvenile and adult leaves, but also involving architecture, anatomy, physiology, and ecological strategies [32,34]. Vegetative phase change, widely discussed by Poethig, represents a process regulated by internal temporal pathways, including mechanisms associated with microRNAs, physiological age, hormones, and endogenous signals [31,44,52,53,54].

These transitions do not only produce morphological modifications. They may also alter the metabolic competence of tissues and organs, reorganizing the biosynthesis, allocation, and compartmentalization of terpenoids, alkaloids, phenolics, glucosinolates, cyanogenic compounds, and other classes of specialized metabolites. Studies of cyanogenic *Eucalyptus* have demonstrated temporal trajectories of chemical defense throughout development, indicating that defensive investment accompanies specific ontogenetic transitions [39]. Similarly, ontogenetic changes in the chemical profiles of *Piper* species indicate that distinct developmental stages may possess their own metabolic signatures, with implications for chemotaxonomy, chemophenetics, pharmacognosy, and bioprospecting [19].

Tissue differentiation represents another decisive level. Specialized metabolism is frequently tissue-specific or cell-specific, being regulated by trichomes, idioblasts, ducts, cavities, laticifers, epidermis, specialized parenchyma, or vascular tissues. Therefore, chemical ontogeny should not be interpreted only at the scale of the whole organism, but also at organographic, histological, and cellular scales. This approach is essential for understanding why different parts of the same plant may present contrasting chemical profiles, even when collected at the same time and under the same environmental conditions.

Chemical ontogeny should be understood within a chemophenetic approach. Chemophenetics, as proposed by Ramos et al. [4], may be understood as an integrative field devoted to the analysis of chemical phenotype variation across different spatial, temporal, ecological, evolutionary, and organizational scales. It is not limited to comparisons among species, nor to the identification of stable chemical markers. On the contrary, it seeks to understand how chemical profiles emerge, vary, become structured, and acquire biological meaning in cells, tissues, organs, individuals, populations, species, and lineages.

Within this framework, chemical ontogeny does not constitute a field parallel to chemophenetics, but rather one of its fundamental dimensions. It corresponds to the temporal-developmental dimension of chemophenetic variation, that is, to the trajectory of transformation of the chemical profile throughout the life of the organism, organs, and tissues. Chemical phenotypic plasticity represents a related, but distinct, dimension, whose expression may be conditioned by the developmental state of the organism [4].

Chemodiversity occupies a complementary position within this conceptual system. If chemophenetics constitutes the interpretative field that analyzes variation in chemical phenotypes, chemodiversity represents the descriptive, quantitative, and organizational dimension of this variation. It allows the measurement of richness, abundance, distribution, redundancy, uniqueness, composition, distance, and structure of metabolites in a given biological system [55]. Therefore, chemical phenotypic plasticity helps explain why chemical profiles vary; chemical ontogeny delimits when and along which developmental trajectory this variation occurs; and chemodiversity allows one to describe and quantify how this variation is organized in chemical, biological, and ecological space.

This organization prevents undue overlap among field, process, trajectory, and pattern: chemophenetics provides the integrative field; chemical phenotypic plasticity describes the capacity for environmentally responsive chemical expression; chemical ontogeny delineates the temporal-developmental trajectory of the chemical phenotype; and chemodiversity describes and quantifies the resulting metabolic patterns [4].

The distinction between developmental phase and developmental stage is useful for organizing this discussion. The term *developmental phase* may be used to designate a broader phase of the ontogenetic trajectory, such as the juvenile, adult vegetative, reproductive, fruiting, or senescent phase. It is a broad biological category related to the overall state of the individual, vegetative phase change, plant architecture, and the position of the organism within its ontogenetic trajectory [31,32,33,52]. In contrast, the term *developmental stage* is more appropriate when indicating a more delimited operational unit within a descriptive, experimental, or phenological scale, such as the newly expanded leaf stage, floral bud stage, anthesis stage, green fruit stage, fruit-ripening stage, or leaf-senescence stage. This meaning is close to the usage adopted in standardized phenological scales, such as the BBCH scale, which codes growth and developmental stages based on observable morphological criteria [56,57]. Thus, the phase indicates the broad position of the plant in its life cycle, whereas the stage delimits a more precise condition of an organ, tissue, or morphophysiological event.

This distinction is fundamental because specialized metabolism may vary simultaneously across the ontogenetic condition of the whole individual and the developmental condition of each organ. For example, a plant in the reproductive phase may simultaneously contain expanding and mature leaves, flowers at anthesis, immature fruits, and senescent tissues, each with distinct metabolic competence. Consequently, it is not sufficient to state that a sample was collected from an adult plant or from a plant in the reproductive phase; it is necessary to indicate the developmental stage of the analyzed organ, its position on the plant, relative age, functional condition, and, whenever possible, degree of anatomical differentiation. This precision prevents chemical differences attributed to the “plant phase” from actually reflecting local organ maturation or tissue-specific differentiation, since plant development is regulated spatially and temporally at different levels of organization [1,2,3,33]. This interpretation is also consistent with multiscale approaches to plant architecture [33].

At this differentiated cellular and tissue substrate, chemical phenotypic plasticity acquires distinct developmental limits and possibilities. Young, expanding tissues frequently exhibit intense biosynthetic activity, greater demand for protection, and high sensitivity to hormonal and environmental signals. Mature tissues, in contrast, may show greater functional specialization, stronger metabolite compartmentalization, fully differentiated secretory structures, and comparatively stable chemical profiles. Senescent tissues may undergo degradation, nutrient remobilization, metabolite oxidation, changes in defense, and shifts in the balance between synthesis and catabolism [58]. Consequently, the developmental state of a tissue may condition not only its metabolic profile but also the magnitude and direction of its plastic response to environmental variation [3,59].

Beyond the distinction between ontogeny and phenology outlined above, methodological studies must also separate whole-plant ontogenetic transitions, local organ maturation, seasonal environmental variation, and chemical phenotypic plasticity. Organ maturation refers to the local trajectory of differentiation and functionalization of a specific structure, such as a leaf, flower, fruit, seed, or root. Chemical phenotypic plasticity refers to the capacity of a genotype to express different chemical phenotypes in response to variable environmental conditions, with the magnitude and direction of this response potentially depending on developmental context [2,4].

These categories may coexist, but they should not be treated as equivalent. A difference in terpene content between young and mature leaves may be associated with ontogeny when it consistently follows organ age or developmental stage. The same difference may reflect seasonality when collections occur under contrasting climatic conditions, phenology when sampling is linked to flowering or fruiting, or environmental plasticity when induced by herbivory, light availability, pathogens, or water deficit. Under natural conditions, these dimensions frequently overlap, making it methodologically unsafe to infer ontogenetic causality without an appropriate experimental design [60].

For this reason, the inference of ontogenetic chemical variation requires explicit control or modeling of environmental and seasonal variables. Greenhouse studies, common gardens, clones, time series, repeated sampling, photoperiod control, known organ age, and multivariate analyses may help separate development, environment, and seasonality. In the field, when these variables cannot be fully controlled, it is more prudent to state that a given variation is associated with ontogeny, rather than that it is exclusively determined by it.

This caution has direct implications for the pharmacognosy, bioprospecting, and quality control of botanical raw materials. In medicinal plants, even subtle developmental differences may alter the occurrence, content, proportion, and stability of bioactive metabolites. Therefore, the description of plant material should include not only species identity, sampled organ, and collection site, but also the developmental phase of the plant, developmental stage and relative age of the organ, phenological condition, organ position, cultivation environment, seasonal timing, and postharvest processing [4].

Incorporating these variables increases robustness in chemical marker selection, determination of optimal harvest time, standardization of herbal drugs, interpretation of bioassays, and comparison among phytochemical studies. It also reduces the risk that differences resulting from tissue age, organ maturation, phenological condition, or environmental exposure will be erroneously interpreted as chemotypes, taxonomic variation, or stable diagnostic markers. Chemical ontogeny thus provides an indispensable methodological foundation for the chemophenetic analysis of plant metabolic diversity whenever chemical composition has pharmacological, ecological, taxonomic, or technological relevance [4].

### 2.2. Developmental Drivers of Specialized Metabolism

The expression *developmental drivers of specialized metabolism* can be understood as a conceptual key for interpreting the production of specialized metabolites not as a peripheral, accessory, or merely “secondary” phenomenon, but as the result of ontogenetic dynamics that organize, modulate, and spatialize plant chemistry throughout development [61,62,63].

Plant ontogeny represents a sequence of regulated transitions in which meristems, secretory structures, leaves, stems, roots, flowers, and fruits acquire distinct metabolic competences. In this context, phase changes do not correspond only to morphological alterations, but also to the reprogramming of biosynthetic capacity. The *miR156-SPL* pathway constitutes one of the most robust examples of this connection among age, vegetative phase, reproductive transition, and specialized metabolism. In *Arabidopsis thaliana* (L.) Heynh. (Brassicaceae), SPL transcription factors regulated by *miR156* negatively modulate anthocyanin biosynthesis, demonstrating that the accumulation of these flavonoids is coordinated with plant aging and phase transition [64]. This axis was subsequently expanded by the demonstration that the *miR156-SPL9-DFR* pathway articulates development and tolerance to abiotic stress, connecting physiological age, environmental response, and phenylpropanoid metabolism [65]. In woody species, such as *Populus* L. (*Salicaceae*), the *miR156* module also regulates anthocyanin biosynthesis through SPL targets and interactions with other microRNAs, reinforcing its functional conservation across different plant lineages [66].

This perspective allows chemical phenotypic plasticity to be understood as an emergent property of developmental regulation. The plant does not produce the same chemical repertoire at every moment of its life; rather, each ontogenetic phase establishes windows of metabolic competence. In grapevine fruits under water deficit, for example, *miR156b* and its targets *VvSBP8/13* act downstream of the abscisic acid signal, regulating anthocyanin biosynthesis in response to drought [67]. This type of evidence indicates that ontogeny should not be treated merely as a chronological variable, but as a regulatory matrix that defines tissue sensitivity to hormonal and environmental signals. Therefore, the terminology developmental drivers is conceptually appropriate because it designates factors that drive, restrict, or redirect metabolic programs as a function of plant age, organ, tissue, and physiological state.

Hormonal networks constitute a central interface between development and specialized metabolism. Jasmonates, salicylic acid, abscisic acid, ethylene, auxins, cytokinins, gibberellins, and brassinosteroids do not act as isolated signals; rather, they compose integrated circuits that adjust growth, defense, differentiation, and metabolic allocation. Nicotine biosynthesis in tobacco is a classical model of this integration. Jasmonate-induced nicotine formation depends on components such as COI1 and JAZ, whereas transcription factors associated with the *NIC2* locus, especially ERFs, regulate structural genes of the biosynthetic pathway [68,69]. Subsequently, MYC2 was shown to regulate nicotine biosynthetic genes both directly and through ERFs of the *NIC2* locus, establishing a hierarchical architecture of jasmonate-induced transcriptional control [70]. This model demonstrates that specialized metabolites may be produced by highly coordinated hormonal regulons, in which signal perception, transcriptional repression/derepression, and activation of biosynthetic genes are temporally integrated.

Recent advances have broadened this interpretation by showing that MYC2 acts as a broad regulatory switch connecting development, stress response, and the synthesis of different classes of specialized metabolites [71,72]. In addition, brassinosteroids participate in the regulation of vegetative phase change in interaction with the age pathway, reinforcing that plant development is governed by a logic of convergence between endogenous signals and genetic programs [73]. Protein kinases associated with specialized metabolites have also been proposed as components of metabolic reprogramming, suggesting that post-translational regulation should be incorporated into the understanding of ontogenetic drivers [74]. Thus, the production of specialized metabolites depends not only on the presence of pathway genes, but also on the contextual activation of these genes by hormonal networks, transcription factors, microRNAs, chromatin, kinases, and environmental signals.

Plant metabolic specialization is inseparable from cell differentiation. Tissues and organs do not merely accumulate compounds at different intensities; they frequently distribute biosynthetic steps among specific cell types. Glandular trichomes, laticifers, idioblasts, epidermal cells, specialized parenchyma, secretory ducts, and vascular structures may function as anatomical units of chemical production, storage, transport, or defense. Glandular trichomes are paradigmatic examples, as they combine high biosynthetic capacity, subcellular compartmentalization, and accumulation of terpenoids, phenylpropanoids, acyl sugars, and other metabolites of ecological and biotechnological interest [75,76,77]. Regulation by MYB and bHLH factors demonstrates that the development of secretory cells and the activation of specialized pathways are coordinated processes, not independent events [78].

This coordination has relevant methodological consequences. Samples obtained from whole organs may mask critical differences among cell types, diluting localized transcriptional and metabolic signals. For this reason, single-cell, spatial transcriptomic, and multi-omics approaches have redefined the analysis of specialized metabolism. In *Catharanthus roseus* (L.) G. Don (Apocynaceae), single-cell RNA-Seq studies have provided a high-resolution map of the multicellular compartmentalization of monoterpene indole alkaloid biosynthesis, showing that the pathway cannot be fully understood without considering cell identity and the spatial distribution of biosynthetic genes [79,80]. Subsequent studies have demonstrated cell-type-dependent regulatory landscapes and specific regulons related to the biosynthesis of these alkaloids, including *feedback* and *feedforward* activation circuits [81,82].

Compartmentalization represents another fundamental conceptual axis. Traditionally, the organization of biosynthetic pathways has been interpreted at the subcellular scale, considering plastids, endoplasmic reticulum, vacuoles, mitochondria, peroxisomes, and cytosol as compartments with distinct chemical microenvironments. This view remains essential, as many natural metabolites depend on spatial separation among enzymes, reactive intermediates, cofactors, pH, redox potential, and transport systems [83]. However, recent literature has shown that compartmentalization also occurs at the multicellular scale, in which different steps of the same pathway are distributed among distinct cells or tissues, requiring the intercellular transport of intermediates [84,85].

In *Catharanthus roseus*, the biosynthesis of strictosidine and monoterpene indole alkaloids involves subcellular organization as well as trans-tonoplast and intercellular transport of intermediates, demonstrating that the pathway is spatially fragmented and functionally integrated [84,86]. In *Arabidopsis*, organ-specific glucosinolate profiles are established through the integration of biosynthesis and long-distance transport, showing that the chemical composition of an organ may depend on metabolic and vascular processes distributed throughout the plant body [87]. This logic redefines the notion of a “biosynthetic pathway”: a pathway is not merely a linear sequence of enzymatic reactions, but a spatialized network of cells, organelles, transporters, anatomical barriers, and developmental gradients. For this reason, the vacuolar transportome and intercellular transport systems should be considered constitutive parts of specialized metabolism regulation, and not merely as accessory mechanisms of storage or detoxification [85,88].

The integration between ontogeny and environment constitutes the point of convergence of the drivers of specialized metabolism. The chemical response of a plant to water stress, herbivory, radiation, temperature, microorganisms, or nutrient availability depends on the developmental stage, the organ analyzed, cellular competence, and physiological history. Thus, environmental signals do not act on an abstract plant, but on an organism situated within a specific ontogenetic phase. This dependence explains why the same stimulus may produce different metabolic responses in seedlings, juvenile leaves, mature leaves, flowers, fruits, roots, or senescent tissues. Specialized metabolites act in defense, signaling, pollinator attraction, plant–microorganism interactions, and stress tolerance, but these functions are modulated by developmental priorities and *trade-offs* among growth, reproduction, and protection [89,90]. The ecological consequences of these developmental changes are represented in Figure 1, particularly in the feedback between chemical traits, biotic interactions, defense, attraction, adaptation, and plant life history.

Consequently, the contemporary understanding of specialized metabolism requires approaches resolved in time and space. The simple quantification of compounds at a single point in development is insufficient to explain biosynthetic dynamics. It is necessary to integrate genomics, epigenomics, transcriptomics, proteomics, metabolomics, anatomy, chemical imaging, and environmental data into experimental designs that consider ontogenetic phase, cell type, organ, tissue, seasonality, and ecological condition. This *multilayered* perspective shows that specialized metabolism is the product of a layered regulatory architecture involving genes, chromatin, regulatory RNAs, transcription factors, hormones, kinases, transporters, organelles, cells, tissues, organs, and the environment [62,63,91,92,93]. Thus, *developmental drivers* constitute the mechanistic backbone of the manuscript because they allow plant chemodiversity to be interpreted as an ontogenetic, spatially compartmentalized, and environmentally responsive phenomenon. More than a descriptive category, “specialized metabolism” becomes an explanatory terminology: it designates the capacity of plants to produce chemical diversity through the integration of development, cell differentiation, molecular regulation, and ecological experience [90,91,92,93,94,95,96,97,98,99,100].

This interpretation is particularly relevant for genera rich in specialized metabolites, such as *Piper* L. (Piperaceae), in which chemical variation cannot be attributed solely to the taxonomic identity of the species, but must be understood as the result of the interaction among ontogenetic stage, analyzed organ, phenology, microclimate, and ecological context (Table S2). Gaia et al. [19], when evaluating different *Piper* species, demonstrated that seedlings and adult plants present markedly distinct chemical profiles using an integrated approach based on ^1^H NMR, principal component analysis, HPLC-DAD, HPLC-HRESIMS, and GC-MS. These results reinforce that ontogeny acts as a structuring force of metabolic composition, qualitatively and quantitatively modulating the accumulation of specialized metabolites throughout plant development [19]. Complementarily, De Brito-Machado et al. [60], in a study with *Piper mollicomum* Kunth, demonstrated that the volatile composition of essential oils varies between leaves and reproductive organs during the reproductive period, in association with phenological and microclimatic information and insect visitation (Table S2). The study used hydrodistillation to obtain essential oils and GC-MS and GC-FID analyses for the identification and quantification of volatile constituents, evidencing that metabolic specialization should be analyzed as a temporal, ecological, and organ-specific phenomenon [60]. These empirical findings support the central premise of this section: specialized metabolites are products of a dynamic regulatory architecture in which development, environment, and ecological interactions converge to produce functionally situated chemodiversity.

### 2.3. Ontogenetic Trajectories of Specialized Metabolites Across the Plant Life Cycle

The diversity of patterns described in this section is summarized in Table S2 [17,60,82,92,101,102,103,104,105,106,107,108,109,110,111,112,113,114,115,116,117,118,119,120,121,122,123,124,125,126,127,128,129,130,131,132,133,134,135,136,137,138,139,140,141,142,143,144,145,146,147,148,149,150,151,152,153], which presents representative examples reported between 2021 and 2026. Across seeds, seedlings, vegetative organs, reproductive structures, fruits, and senescent tissues, specialized metabolism follows distinct trajectories associated with defense, signaling, pollinator attraction, dispersal, storage, maturation, and pharmacognostic quality. These trajectories reflect the continuous reorganization of metabolite composition, concentration, allocation, and function in response to ontogenetic priorities, resource limitations, and ecological pressures [15,19,39,51,92,93].

Seeds represent a particular stage of plant ontogeny in which the chemical repertoire is directed toward energy storage, defense, and embryo protection during the period of quiescence. In *Arabidopsis thaliana*, dormant seeds contain glucosinolate concentrations that may reach 63 µmol g^−1^ dry weight, with a profile dominated by methylthioalkyl forms and by hydroxylated and benzoylated derivatives that are largely absent from vegetative tissues [94,95]. In *Camelina sativa* (L.) Crantz (*Brassicaceae*), long-chain glucosinolates, especially C8–C11 compounds, preferentially accumulate in the seed coat and endosperm, whereas short-chain homologues are concentrated in the embryo. Despite differences in biosynthetic capacity among tissues, this segregation appears to be mainly determined by transporter specificity [96]. Phenolic compounds, such as flavonoids, perform functions associated with dormancy and the regulation of events preceding germination, showing differential distribution within seeds. Proanthocyanidins, for example, are deposited in the inner layer of the seed coat, contributing to pigmentation and protection against pathogens [97].

Germination triggers a profound remodeling of this metabolome. In *Arabidopsis*, 4-methylsulfinylbutyl glucosinolate increases during the first days after imbibition, whereas 4-methylthiobutyl glucosinolate, predominant in the seed, may decrease by up to 75% [94]. In legumes, tannins, phytic acid, and trypsin inhibitors decrease during germination, whereas isoflavones and saponins increase, reflecting a metabolic reorientation from seed protection toward seedling establishment [99]. This set of transitions, observed in phylogenetically distant groups, indicates that germination represents a period of active chemical reorganization integrated with the ontogenetic processes that lead to seedling establishment.

With the progressive depletion of cotyledonary reserves and the increasing dependence on photosynthesis, resource allocation to defense begins to compete directly with the demands of biomass accumulation [51]. This metabolic and ecological tension may result in a transient reduction in defensive metabolites during early development, a pattern documented in multiple plant systems [15,39]. In cyanogenic *Eucalyptus yarraensis* Maiden & Cambage (Myrtaceae), prunasin begins at low levels in the cotyledons and progressively increases until reaching a maximum value around 240 days after sowing. The cotyledonary stage was chemically impoverished, possibly because the small seed size does not provide sufficient nitrogen to sustain expressive synthesis immediately after germination [39]. This pattern, however, is not universal. In species with early defense strategies, such as *Catharanthus roseus*, seedlings may accumulate terpenoid indole alkaloids corresponding to approximately 2% of total biomass even before the first true leaves are fully expanded [100].

The nature of early defense varies in magnitude as well as in metabolic and regulatory quality. The jasmonic acid pathway, responsible for a large proportion of herbivory-induced defenses, operates with high responsiveness during juvenile stages, but its amplitude tends to decline with plant age [93]. This example illustrates how ontogenetic processes, including epigenetic mechanisms, contribute to defining the biosynthetic competence expressed under different developmental conditions. The capacity for induced defense does not depend only on the immediate physiological state of the tissue, but is embedded within the plant’s ontogenetic program. Thus, the same herbivore attack may trigger qualitatively distinct molecular responses depending on the age of the organism.

With continued growth, constitutive metabolites tend to increase in vegetative tissues. In several woody species, mature plants present higher concentrations of total phenolics, phenolic glycosides, and resinous compounds than seedlings [51]. In *Arabidopsis*, mature plants maintain higher basal glucosinolate reserves and exhibit greater resistance to chewing insects, although herbivory-induced jasmonic acid pulses are paradoxically lower [93]. This decoupling between induced defense and constitutive defense reflects a strategic shift throughout ontogeny, in which young plants tend to prioritize inducible responses due to the high cost of constitutive defense under limited resources, whereas mature plants sustain basal defenses at higher levels, reducing their dependence on rapid induction after damage [93,95].

During the vegetative phase, the developmental stage of individual leaves introduces an additional metabolic gradient. In *Arabidopsis* rosettes, young leaves located in the center may present glucosinolate concentrations greater than 60 µmol g^−1^ dry weight, whereas older peripheral leaves present values below 10 µmol g^−1^ [94]. A comparable gradient occurs in maize, in which younger leaves accumulate higher concentrations of DIMBOA-Glc and DIM2BOA-Glc, in accordance with optimal defense theory [98]. In seedlings of *Eucalyptus globulus* Labill. (Myrtaceae), total terpene content increased 1.3- to 2.1-fold across the first five pairs of true leaves, although monoterpenes and sesquiterpenes showed divergent dynamics: β-caryophyllene accumulated rapidly in the first leaf pairs before declining, whereas most monoterpenes showed a more gradual increase [154]. The fact that two chemical classes present in the same tissues follow such distinct trajectories suggests that partially independent ontogenetic programs regulate their synthesis, possibly according to differentiated ecological roles within specific growth windows.

The transition to reproductive maturity reconfigures metabolic priorities in a manner frequently associated with reduced investment in vegetative defense. In monocarpic species, allocation to reproduction may occur at the expense of foliar defense; in tomato and tobacco, for example, the inducibility of jasmonic acid-mediated defenses is substantially attenuated or lost after flowering [15,51]. Reproductive organs mobilize specific sets of specialized metabolites adjusted to their ecological functions, including gamete protection, pollinator attraction, mediation of biotic interactions, and defense of developing seeds.

In flowers, specialized metabolism acts simultaneously in protection and pollinator attraction. In Brassicaceae, glycosides of kaempferol, quercetin, and isorhamnetin accumulate in petals, absorbing ultraviolet radiation and structuring nectar-guide patterns perceived by bees. Phenylamides, especially spermidine derivatives, are concentrated in anthers and pollen, where they act as constituents of the pollen coat essential for male fertility [155]. Floral volatile emission follows its own temporal trajectory: it increases with flower opening, reaches a plateau during full anthesis, and ceases after pollination. In night-flowering species, the emission peak coincides with pollinator activity and persists under constant light, indicating regulation by the circadian clock [156]. In *Rhododendron pulchrum* Sweet (Ericaceae), most identified flavonoids reached their highest levels during full flowering, including anthocyanins, flavones, and flavonols, demonstrating that flower opening is accompanied by active and differential accumulation of these chemical classes [115].

Fruits present a distinct ecological challenge, as they must protect developing seeds while simultaneously becoming attractive to animal dispersers. The glucosinolate system in *Ochradenus baccatus* Delile (Resedaceae) illustrates how the same class of metabolites may change function during maturation: glucosinolates present in the pulp react with seed myrosinase during chewing, generating toxic isothiocyanates that deter seed-predating rodents without affecting birds that swallow fruits whole and therefore do not trigger the enzyme–substrate reaction [157]. This mechanism of directed deterrence demonstrates that metabolites present in fruits may function as selective filters within animal communities, favoring mutualistic interactions and discouraging antagonistic ones.

Leaf senescence is marked by the progressive dismantling of cellular components and the mobilization of nutrients to growing or reproductive organs. Specialized metabolites actively participate in this process. In *Arabidopsis* rosette leaves, glucosinolate concentrations decline to approximately 10% of the values observed in mature leaves during senescence and to only 4% in dead leaves [94]. This decline cannot be attributed exclusively to passive catabolism, as it coincides with the development of inflorescences, siliques, and seeds that simultaneously accumulate glucosinolates. In addition, tracer experiments have demonstrated the phloem transport of these molecules from leaves to reproductive organs [94,95], evidencing chemical regulation driven by ontogeny. In mature plants, it has also been proposed that glucosinolates may migrate from senescent leaves to young expanding leaves, preserving a protective gradient along the aerial portion without requiring an immediate additional biosynthetic cost [93].

An exception to the general pattern of metabolic decline during senescence is the accumulation of anthocyanins in deciduous species, responsible for the red and purple colors frequently observed in autumn in temperate regions. This late synthesis is stimulated by nitrogen and phosphorus depletion, low temperatures, and high light intensity, with ethylene, jasmonic acid, and abscisic acid acting as convergent hormonal signals [158].

Beyond the temporal axis, specialized metabolites are heterogeneously distributed among organs and anatomical compartments. Roots display a chemical composition distinct from that observed in aerial organs. Flavonoids secreted by root tips participate in the recruitment of rhizobia and mycorrhizal fungi; coumarins, such as scopoletin, mediate iron acquisition in alkaline soils and modulate rhizosphere microbial communities; and benzoxazinoids released by maize roots exert selective effects on rhizosphere microbial communities, favoring plant growth-promoting taxa and limiting pathogen proliferation [159,160]. The glucosinolate repertoire in *Arabidopsis* roots is considerably more restricted than that observed in aerial parts: only ten compounds have been identified, with a predominance of indolic forms and complete absence of benzylic forms [94,95,160].

Within seeds, the distribution of specialized metabolites is also not uniform. In *Arabidopsis*, proanthocyanidins remain confined to the endothelium of the inner integument, whereas flavonols are distributed among the seed coat, embryo, and endosperm according to their glycosylation state [97]. These patterns reflect the distinct functional roles of each tissue: the seed coat is responsible for external defense and structural protection, whereas the embryo receives compounds that may protect it during germination or provide nitrogen and sulfur during early seedling establishment.

In flowers, metabolic specialization reaches the level of cell types. Phenylamides are concentrated in the stamens, flavonol glycosides accumulate in UV-reflecting zones of the petals, and volatile biosynthesis remains largely restricted to the conical epidermal cells of the corolla, from which emission occurs through passive diffusion and active transport mediated by ABC transporters [155,156]. The confinement of myrosinase to idioblastic cells in vegetative tissues of Brassicaceae, physically separated from vacuoles containing glucosinolates, illustrates a broader principle: the ecological and physiological function of specialized metabolites depends on both subcellular and tissue compartmentalization and absolute ontogenetic expression. Therefore, the ontogenetic trajectory of specialized metabolism cannot be adequately represented by a single temporal curve. It emerges from the interaction of multiple processes, including ontogenetic regulation, spatial compartmentalization, resource availability, and environmental and ecological pressures, configuring a dynamic chemical phenotype throughout the plant life cycle.

### 2.4. Ecological Consequences of Chemical Ontogeny

Chemical ontogeny acquires ecological significance when stage-dependent chemistry alters the probability or outcome of interactions with herbivores, mutualists, dispersers, or microorganisms. Because developmental stages differ in resource allocation, vulnerability, and functional priorities, resistance and tolerance strategies may change gradually or abruptly across the life cycle, including recurrent increases or decreases in defensive metabolites [15,16,40,51,161].

Evidence obtained from different plant species supports this ontogenetic pattern. Goodger, Choo, and Woodrow [39], studying *Eucalyptus froggattii* Blakely (Myrtaceae), observed higher concentrations of cyanogenic compounds in young tissues and a gradual reduction in mature leaves. In *Nicotiana attenuata* Torr. ex S.Watson (Solanaceae), changes in the production of nicotine and volatile compounds modify both resistance against herbivores and the attraction of natural predators [162,163].

The distribution of specialized metabolites among organs and tissues reflects both optimal defense and developmental trade-offs. Structures of greater adaptive value or vulnerability tend to receive greater defensive investment, but this allocation competes with growth, reproduction, and metabolic maintenance [51,164,165,166]. Accordingly, seedlings and juveniles often balance rapid growth with protection against herbivory, whereas adults increasingly allocate resources to flowering and fruit production. In *Populus tremuloides* Michx. (Salicaceae), salicinoid phenolic glycosides and condensed tannins decrease during the juvenile phase and stabilize at maturity, demonstrating how ontogenetic defense trajectories may affect individual fitness and population-level ecological patterns [167,168,169,170].

Ontogenetic changes also affect mutualistic interactions involving pollinators and seed dispersers. Floral scent, nectar, and other specialized metabolites act as chemical signals and ecological filters whose composition and emission vary with reproductive development [171,172,173,174,175]. Young, reproductively viable flowers may exhibit stronger volatile emission, whereas ripening fruits begin to release signals associated with nutritional quality and maturation status. Chemical ontogeny therefore links defense, ecological communication, partner selection, seed dispersal, and reproductive success [157,172,176,177].

Another important aspect related to plant ontogeny involves plant–microorganism interactions. Plants live in association with different bacteria, fungi, and other microorganisms present in the rhizosphere and internal tissues, establishing mutualistic or pathogenic relationships. Among the main examples of these associations are nitrogen-fixing bacteria and mycorrhizal fungi, which contribute to nutrient acquisition, protection against pathogens, and increased tolerance to environmental stresses [178,179].

The composition of these microbial communities varies throughout plant ontogeny. Changes in root exudates, root architecture, and tissue chemical composition promote shifts in the structure and functionality of the plant-associated microbiome [178]. Thus, ontogenetic stage is considered an important factor structuring bacterial and fungal communities associated with plants. Leroy et al. [180] observed changes in root traits and in the composition of associated fungi throughout plant development, indicating that different ontogenetic phases favor distinct functional groups of microorganisms. In addition, the assembly of these communities is directly related to plant *fitness*, influencing growth, resistance to pathogens, and environmental adaptation [179].

Incorporating ontogeny into plant functional ecology reveals that individuals of the same species may occupy different ecological roles across development. Ontogenetic changes affect resource acquisition, stress resistance, growth, reproduction, plant architecture, specific leaf area, chemical composition, and interactions with herbivores, pollinators, dispersers, and microorganisms [40,181,182,183]. This interpretation is consistent with Grubb’s *regeneration niche*, according to which developmental phases differ in ecological requirements, and with evidence that plant age contributes substantially to intraspecific functional-trait variation [184].

### 2.5. Analytical Strategies for Studying Chemical Ontogeny

*Developmental windows* are periods in which rapid chemical transitions or stage-contingent responses occur over relatively short intervals [185,186,187]. Because chemistry from mature plants cannot be assumed to represent seedlings or juvenile individuals, experimental designs must define developmental stages using explicit morphological and biological criteria, adopt sufficient temporal resolution, and stratify samples by organ, tissue, position, and age. These measures are essential for capturing metabolic peaks and avoiding the masking of biologically relevant variation [183,188,189,190,191,192,193,194,195].

Given the high complexity and dynamic nature of metabolism associated with plant development, metabolomics has become one of the main approaches for investigating chemical ontogeny as it enables the simultaneous analysis of diverse metabolites [196]. It may be targeted, as in *targeted metabolomics*, with a focus on precise quantification, allowing for the monitoring of metabolites previously associated with specific physiological processes [197,198], or untargeted, as in *untargeted metabolomics*, with an exploratory character aimed at discovering new compounds and unexpected chemical transitions among stages [199,200]. In the context of chemical ontogeny, the choice depends on prior knowledge of the system and the biological objectives of the investigation. These approaches are complementary and may be integrated, supporting both the discovery and validation of ontogenetic patterns, always accompanied by experimental and statistical rigor [201,202,203].

Analytical platforms include LC-MS, GC-MS, and NMR, each with specific advantages in covering the plant metabolome [204,205]. The choice of analytical platform directly influences which dimensions of chemical ontogeny can be observed. GC-MS is more suitable for volatile compounds and primary metabolites, whereas LC-MS covers more complex and non-volatile metabolites and is central to untargeted metabolomics [196,206]. NMR provides complementary information with high reproducibility and structural confirmation [207]. Similarly, MALDI and mass spectrometry imaging technologies have become increasingly important because they add spatial information to the metabolomic analysis of plant development [208,209]. The integration of multiple platforms and complementary techniques, such as FT-ICR-MS, IMS-MS, and imaging, expands metabolite coverage and spatial interpretation [208,210].

Metabolomic studies generate large datasets that through the use of chemometrics, can be transformed into interpretable biological representations. Since hundreds or thousands of metabolites may vary concomitantly throughout development, multivariate approaches offer substantial advantages by enabling the identification of emerging patterns associated with ontogenetic transitions [204,211,212,213,214,215,216,217,218,219,220]. In this scenario, principal component analysis, or *PCA*, represents an important exploratory strategy by revealing global trends and identifying clusters associated with different developmental stages without the prior imposition of experimental categories [212].

Supervised methods, such as *Partial Least Squares Discriminant Analysis* (PLS-DA) and *Orthogonal Partial Least Squares Discriminant Analysis* (OPLS-DA), are widely used to identify metabolites associated with specific developmental events, including maturation, reproduction, and senescence, thereby enabling the reconstruction of metabolic trajectories [204]. However, the high number of variables characteristic of metabolomic datasets increases the risk of overfitting, making rigorous validation procedures indispensable. Under natural conditions, development-related signals frequently overlap with environmental, seasonal, and physiological variability, making it difficult to distinguish ontogenetic effects from other sources of variation. For this reason, longitudinal analyses and methods aimed at reconstructing temporal trajectories have gained relevance. In this context, machine-learning approaches have expanded the capacity to detect complex and nonlinear relationships between metabolism and ontogeny [211].

Metabolic reorganization may become evident before individual compounds are identified. Modern LC-MS/MS platforms detect thousands of signals, but only a fraction can be confidently assigned to known structures, creating an *annotation bottleneck* that is especially consequential when metabolites occur only within specific developmental windows, organs, or tissues [213,214,215,216]. Identification-independent approaches therefore use spectral relationships, chemical substructures, and coordinated abundance patterns to retain biologically informative, unannotated signals rather than treating them as noise [213,214,220].

Molecular networking addresses this limitation by representing similarities among MS/MS fragmentation spectra as structural relationships among compounds. In chemical ontogeny, these networks can reveal coordinated expansion, replacement, or reduction in metabolic families across developmental stages without requiring complete prior identification, although spectral similarity must not be interpreted as definitive structural identity [215].

The consolidation of the *Global Natural Products Social Molecular Networking* (GNPS) platform by Wang et al. [215] represented a milestone for the application of these approaches by enabling the systematic comparison of experimental spectra with databases shared by the scientific community. Subsequently, the development of *Feature-Based Molecular Networking* (FBMN) expanded this capacity by integrating chromatographic information, relative abundances, and signal alignment into molecular networks [216]. As a result, processes of chemical diversification throughout development can be followed at higher resolution, while the possibilities for annotation through collaborative databases are broadened.

In addition to facilitating the visualization of metabolic organization, molecular networks have assumed a substantial role in modern dereplication. Traditionally, dereplication was used to identify previously known compounds at an early stage and avoid redundant efforts in isolation and structural characterization. Currently, the concept has become broader and includes the identification of chemical families, biosynthetic patterns, and regions of chemical space potentially associated with new metabolites [217]. Complementary tools, such as *NPClassifier*, allow inferences about chemical classes and biosynthetic relationships, whereas *Ion Identity Molecular Networking* has reduced redundancies associated with different ionic forms of the same molecule, increasing the interpretative resolution of metabolomic datasets [218,219].

For a long time, unidentified signals were treated as a kind of interpretative noise: useful information for discriminating samples, but insufficient to support broader biological hypotheses. Even when chemical relationships can be inferred, spectral similarity does not always correspond to the structural identity of metabolites. This problem was already evident in approaches such as MS2LDA, proposed by van der Hooft et al. [213], and has gained a new dimension with recent methods of spectral similarity and *identification-free* analysis [214,220].

In identification-independent metabolomics approaches, rather than treating unannotated metabolites as gaps to be discarded, these strategies leverage spectral patterns, inferred structural relationships, and shared metabolic behaviors. The question is no longer only “what is the name of this compound?”, but also “which signals is it related to?”, “at which stage does it appear?”, and “what kind of structural or biosynthetic information can be extracted even before complete identification?”.

Despite these advances, both chemometrics and molecular networks share a fundamental challenge: adequately representing the dynamic and continuous nature of plant development. Spectral similarity does not necessarily equal structural similarity, and statistical relationships do not always reflect causal biological mechanisms. In this context, integration with transcriptomics, genomics, proteomics, and phenomics has become a fundamental strategy for investigating chemical ontogeny [221,222]. Whereas transcriptomics allows metabolic alterations to be related to gene-expression programs and specific biosynthetic pathways, genomics helps identify the biosynthetic potential of species, proteomics brings the analysis closer to the effective metabolic capacity of tissues, and phenomics connects chemical changes to observable morphological and physiological traits. Together, these approaches represent a significant scientific advance in the comprehensive understanding of metabolic dynamics to connect metabolomic patterns to the biological mechanisms that produce them.

From an analytical standpoint, multi-omics integration requires methods capable of relating heterogeneous datasets produced at different scales, such as O2PLS and other integrative modeling strategies, favoring a broader understanding of the regulatory mechanisms underlying the chemical changes observed during development [221,222]. Studies in *Catharanthus roseus*, for example, have demonstrated that integration among genomics, transcriptomics, and metabolomics can reveal the cellular organization of complex biosynthetic pathways, evidencing that the chemical variation observed during development results from the interaction among molecular regulation, cellular specialization, and tissue function [80].

Much of the knowledge on chemical ontogeny has been built from analyses of whole organs or homogenized tissues, approaches that frequently obscure the spatial and cellular organization of plant metabolism. However, recent evidence obtained through spatial metabolomics, mass spectrometry imaging or MSI, and single-cell transcriptomics demonstrates that many processes of specialized metabolism are organized at finer anatomical scales, involving specific cell types and distinct tissue compartments [79,223,224,225]. This perspective is particularly relevant to chemical ontogeny because changes observed throughout development may reflect not only alterations in biosynthetic regulation, but also processes of cell differentiation, the formation of secretory structures, and the functional reorganization of tissues.

In this context, MSI enables metabolites to be mapped directly in tissues while preserving their anatomical organization, whereas single-cell transcriptomics identifies specific cell populations associated with the production of specialized metabolites [79]. The integration of these strategies with other omics approaches has revealed the cellular compartmentalization of complex biosynthetic pathways and allowed metabolomic patterns to be related to developmental processes, tissue differentiation, and the functional specialization of organs [80]. Despite challenges related to cost, infrastructure, analytical resolution, and data integration, spatial and single-cell omics represent an important conceptual shift for chemical ontogeny because they simultaneously incorporate the temporal, spatial, and cellular dimensions of metabolic activity [81,226].

**Figure 2:**
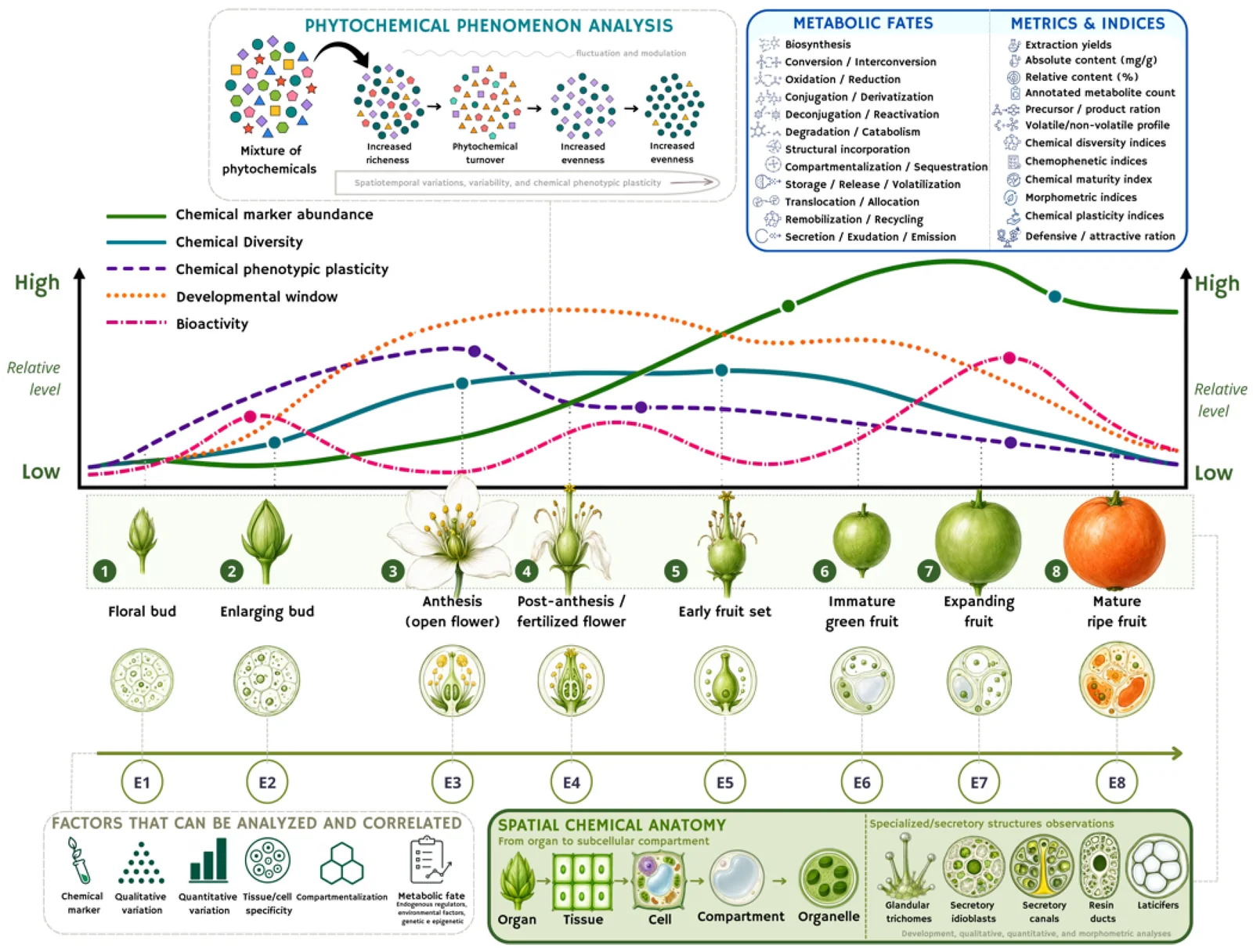
Analytical framework for investigating chemical ontogeny across reproductive development.

Figure 2 presents a theoretical and experimental framework for interpreting chemical ontogeny across plant organs and developmental stages. Floral bud, anthesis, post-anthesis, green fruit, expanding fruit, and mature fruit are used as an illustrative sequence, but the same logic applies to leaves, stems, roots, seeds, and secretory structures at different degrees of differentiation. Each stage is treated as a *chemical-ontogenetic unit* emerging from the interaction among morphoanatomical differentiation, biosynthetic competence, resource allocation, storage, transformation, emission, degradation, and ecological function [1,3,4,7,15,37,38,50]. The trajectories of marker abundance, chemodiversity, chemical phenotypic plasticity, developmental windows, and bioactivity are hypothetical rather than universal; their maxima may occur at different stages, precluding a single, universally optimal collection time.

As empirical examples of these trajectories, De Brito-Machado et al. [60] demonstrated, in *Piper mollicomum* Kunth, a progressive reorganization of essential oils throughout reproductive development. Linalool, 1,8-cineole, and *E-nerolidol* increased during floral maturation and anthesis and decreased during fruiting stages, whereas α-pinene showed a progressive reduction along the ontogenetic sequence. Chemical profiles also shifted from greater similarity at the early stages, mainly associated with linalool, to greater differentiation at the final stages, with a stronger contribution of 1,8-cineole. Complementarily, Felisberto et al. [92] observed, in *Piper rivinoides* Kunth, a chemical transition across five ontogenetic phases of the plant: young individuals in phases I and II showed profiles dominated by the arylpropanoids apiol (59.59–74.69%) and dillapiole (2.29–2.76%), whereas phases III–V were characterized by an increased contribution of monoterpenes, mainly α-pinene (20.03–35.09%), β-pinene (11.45–16.72%), and δ-2-carene (0.32–42.07%). These studies demonstrate, at different scales of plant development, that ontogeny can promote quantitative and compositional changes, as well as a reorganization in the predominance of chemical classes and chemodiversity.

In an experimental application, this variation can be evaluated by GC-MS, LC-MS/MS, UHPLC-DAD, NMR, *untargeted metabolomics*, *molecular networking*, chemical imaging, spatial metabolomics, and approaches integrated with anatomy, histochemistry, and spatial transcriptomics [17,196,215,216,225,226,227,228,229,230]. In the illustrated example, the early stages, organogenesis and differentiation of secretory structures, may be associated with the establishment of biosynthetic competence and the accumulation of precursors and intermediates [1,3,50]. During anthesis, volatile emission, pigment synthesis, and the accumulation of metabolites related to attraction, defense, and ecological communication may intensify [8,9,144,230,231,232,233,234,235,236,237]. After fertilization, metabolic reorganization accompanies embryo protection, fruit growth and maturation, and seed defense, without implying that these trajectories are universal [12,13,16,51].

This distinction is important for pharmacognostic and metabolomic interpretation because a stage with a high concentration of a few dominant compounds may differ fundamentally from one containing a larger number of metabolites at lower proportions. Chemical richness, diversity, marker dominance, and bioactivity are therefore complementary but non-equivalent dimensions [4,6,7,9,48,55,229,231,232,233]. Likewise, a higher marker concentration does not necessarily imply greater bioactivity, which may depend on synergism, antagonism, minor metabolites, oxidation state, conjugation, compartmentalization, and bioavailability.

From an evaluative standpoint, Figure 2 can also be used as a basis to exemplify how ontogenetic variation may be transformed into measurable processes through indices. Chemical richness would correspond to the number of annotated or identified metabolites at each stage. Chemical diversity may be estimated using the Shannon index, in which *p_i_* represents the relative proportion of each metabolite. Dominance or inverse diversity may be assessed using the Simpson index. Pielou’s evenness allows for verification of whether the chemical composition is homogeneously distributed among metabolites or dominated by a few major compounds [4,55,229,235,236,237]. In addition to these classical indices, ontogenetic chemodiversity may be evaluated using dissimilarity metrics, such as Bray–Curtis, Jaccard, Euclidean distance, Manhattan distance, Mahalanobis distance, and Rao’s quadratic diversity [55,229,238]. In addition, redox variations can be evaluated using the ecological redox index, the Ramos & Moreira index [4].

Chemical phenotypic plasticity may be operationalized through the coefficient of variation among stages, calculated as the standard deviation divided by the mean and multiplied by 100. It may also be estimated using the relative phenotypic plasticity index adapted from Valladares, in which the absolute difference between two values is divided by the sum of those values. In a comparison between floral bud and anthesis, or between young leaf and adult leaf, the plasticity of a specific metabolite may be calculated as the absolute value of the difference between the two stages divided by the sum of the observed values. Values close to 1 indicate high relative plasticity, whereas values close to 0 indicate greater chemical stability. This metric may be applied to isolated metabolites, chemical classes, biosynthetic ratios, or multivariate scores derived from PCA, PLS-DA, or principal coordinate analyses [239,240,241].

To connect chemistry and metabolic processes, Figure 2 also makes it possible to exemplify the construction of specific interpretative indices. The precursor/product ratio may indicate biosynthetic advancement or metabolic conversion. The product/precursor ratio may be used as an index of biosynthetic conversion. The oxidized metabolite/reduced metabolite ratio may estimate trends of oxidation, senescence, or redox modulation. The conjugated metabolite/free metabolite ratio may indicate storage, detoxification, or the modulation of bioactivity. The volatile/non-volatile ratio may represent the transition among emission, signaling, local defense, and storage.

The defense/attraction ratio may be calculated as the relative sum of defensive metabolites divided by the relative sum of metabolites associated with pollinator or disperser attraction. In any application, these indices must be explicitly defined according to the hypothesis, metabolic class, organ studied, and ecological, pharmacognostic, or biotechnological purpose [9,75,88,230].

Integration with plant anatomy substantially refines this evaluation. In an applied study, each stage may be characterized by quantitative anatomical variables, such as glandular trichome density per square millimeter, proportion of young, mature, and senescent trichomes, area of secretory cavities, duct diameter, estimated lumen volume, epidermal thickness, proportion of secretory parenchyma, degree of lignification, histochemical intensity, and spatial distribution of metabolites in tissues.

These variables may compose a Secretory Structure Development Index, constructed by z-score standardization of anatomical variables and subsequent weighted combination according to their biological relevance. This index would make it possible to verify whether the increase in chemical markers results from higher biosynthesis per cell, a greater number of secretory structures, greater maturity of these structures, or higher storage capacity (Figure 2).

Another index derived from this approach is the Chemical Compartmentalization Index, defined as the proportion of the chemical signal located in tissues or secretory structures relative to the total chemical signal of the organ. This index may be estimated by quantitative histochemistry, MALDI-MSI, DESI-MSI, Raman, FTIR imaging, or spatial metabolomics. When associated with chromatographic data, it allows diffuse metabolite accumulation in the organ to be differentiated from specific concentration in anatomical compartments. Complementarily, the Tissue Localization Index may compare the abundance of a metabolite in the epidermis, mesophyll, parenchyma, vascular tissue, trichomes, cavities, or laticifers, allowing for interpretation of whether the ontogenetic change is global or tissue-specific [75,76,79,80,88].

Correlations between anatomy and chemistry are fundamental in an experimental application of this model. The correlation between glandular trichome density and terpenoid content may indicate an association between secretory differentiation and the accumulation of volatile metabolites. The correlation between the area of secretory cavities and the content of resins, essential oils, or lipophilic compounds may indicate increased storage capacity. The correlation between degree of lignification and accumulation of phenolics may suggest a transition toward structural and chemical defense.

These relationships may be tested using Pearson correlation when variables show a normal distribution; Spearman correlation when nonparametric monotonic relationships are present; or linear mixed models when individual, organ, stage, environment, and biological replicate are to be incorporated as fixed or random effects [212,229,242]. It is also possible to evaluate the global coupling between anatomy and chemistry through matrix correlation. The association between these matrices may be tested using a Mantel test, Procrustes analysis, co-inertia analysis, or the RV coefficient. High anatomical–chemical coupling would indicate that the most anatomically different stages are also the most chemically distinct. Low coupling would suggest that chemical variation may be more strongly associated with biosynthetic regulation, transport, degradation, environment, or physiological state than with visible anatomical differentiation.

Together, anatomical, chemical, and multivariate analyses help distinguish increased biosynthesis from secretory-structure differentiation and metabolite redistribution. Their integration allows Figure 2 to function simultaneously as a conceptual scheme and an experimental-planning matrix, anchored in empirical evidence of how chemical-ontogenetic trajectories emerge in real plant systems [212,215,216,242].

### 2.6. Translational Implications for Pharmacognosy, Quality Control, and Bioprospecting

The chemical variability of medicinal plants is a central challenge for pharmacognosy because a dynamic biological matrix must be converted into a reproducible, safe, and standardized pharmaceutical input. Rather than being treated only as analytical noise, this variability should be interpreted as a structuring biological property that informs harvesting, processing, authentication, marker selection, quality control, and bioprospecting [21,243,244]. Pharmacognostic quality therefore cannot be defined solely by taxonomic identity: it must also account for plant and organ developmental stage, tissue differentiation, genotype, environment, physiological history, chemical diversity, phenotypic plasticity, bioactivity, metabolic fate, and tissue-specific accumulation. These variables explain why leaves, roots, flowers, fruits, seeds, stems, and underground organs may differ substantially in the yield and relative proportion of specialized metabolites [21,245].

The concept of an ontogenetic window assumes translational importance. It refers to the developmental interval in which a target substance, chemical class, or metabolic profile of interest reaches greater pharmaceutical, technological, or economic suitability. Harvesting outside this window may produce raw materials with insufficient marker content, excessive batch-to-batch variation, low analytical reproducibility, and reduced therapeutic value of the herbal drug [21,246]. Studies with aromatic plants demonstrate that essential-oil yield and composition vary according to vegetative stage and time after sowing [247], whereas other examples show that the most advantageous harvesting point does not necessarily coincide with the maximum concentration of a single metabolite [248]. Harvest-window selection should therefore integrate chemical content, stability, yield, and intended use.

The selection of chemical markers also needs to be re-evaluated in light of ontogeny. A marker suitable at a specific stage may lose its representativeness at another, whether due to changes in the expression of biosynthetic pathways or the tissue redistribution of metabolites. This limitation disadvantages quality control strategies based exclusively on one or a few constituents, especially in plant matrices where the therapeutic effect depends on multiple constituents and quantitative relationships between chemical classes. Conversely, it supports metabolomic and multivariate approaches capable of capturing the sample’s overall chemical state and distinguishing legitimate physiological variation from processes such as degradation, substitution, or adulteration [21,243].

Traceability should therefore incorporate metadata such as species, botanical voucher, organ, estimated age, phenological stage, origin, environmental conditions, agronomic management, drying, storage, and processing, thus reducing the risk of interpreting ontogenetic variation as adulteration or chemical stability [249,250,251]. The work of Kim et al. [249], using NMR-based metabolomics to distinguish *Ilex paraguariensis* A.St.-Hil. (Aquifoliaceae) from adulterant species, showed that arbutin may act as an adulteration marker because it is present in substitute species and absent from authentic yerba mate. However, even useful markers must be interpreted with caution, since populations, individuals, organs, and ontogenetic phases may present distinct chemical ranges [250]. Spaggiari et al. [251], studying *Rumex usambarensis* (Engl.) Dammer (Polygonaceae), demonstrated an uneven distribution of metabolites among organs, with flowers enriched in flavonoids and leaves and stems characterized by a greater presence of benzopyrans and sesquiterpenes.

These implications also extend to chemotaxonomy and chemophenetics. Chemical profiles obtained from different ontogenetic phases may generate artificial chemical distances and misleading classifications when developmental stage is not controlled. A chemophenetic approach allows the chemical phenotype to be interpreted as a dynamic trajectory, distinguishing intraspecific plasticity, programmed ontogenetic variation, and evolutionary divergence among lineages [4,249,252]. Chemical biodiversity is therefore better understood as a repertoire of development-dependent metabolic trajectories rather than a static inventory of compounds.

In bioprospecting, this perspective expands the search for bioactive metabolites beyond mature plants, traditionally used organs, or fixed collection periods. Different developmental stages may exhibit metabolic peaks, biosynthetic intermediates, or transient profiles of pharmacological and biotechnological interest. In the genus *Piper*, for instance, seedlings and mature individuals display marked differences in chemical composition [19], while multi-omics approaches can reveal metabolites restricted to specific time windows [253,254].

This perspective shifts bioprospecting from an extractive and random logic toward a predictive exploration guided by ecophysiological hypotheses. Identifying vulnerable stages, tissues under selective pressure, or moments of stress response can pinpoint metabolic niches with a higher likelihood of yielding bioactive compounds. Alpha, beta, and gamma chemodiversity indices, as proposed by Kessler and Kalske [255], aid in planning multi-scale sampling by distinguishing chemical diversity within individuals, among individuals, between populations, and across environments. When combined with chemophenetics, these indices enable the selection of organ, age, environment, and phenological phase combinations that hold the greatest potential for chemical innovation [4].

In the domestication of medicinal and aromatic plants, the challenge lies in converting the variability of natural populations into predictable production systems without eliminating the chemodiversity necessary for efficacy, adaptation, and innovation. Agronomic management must be adjusted to the ontogenetic triggers that regulate the production of specialized metabolites, moving beyond the simplified opposition between vegetative growth and chemical defense [256]. The definition of the harvest point should abandon generic calendars and adopt phenological, chemical, and ecophysiological markers. Hazrati et al. [257] reinforce that synchronizing harvest with specific ontogenetic stages is a fundamental condition for the quality of essential oils and bioactive extracts. Similarly, cultivar selection should consider not only high active-compound content, but also stability of the metabolomic trajectory, preservation of Q-markers, and maintenance of chemical performance under different environmental pressures [258,259]. In protected cultivation systems and controlled-environment agriculture, control of photoperiod, nutrition, temperature, irrigation, and floral induction makes it possible to modulate ontogenetic transitions, expand harvest windows, and induce specific metabolite peaks. In these systems, the medicinal plant is managed as a living bioreactor whose pharmacognostic performance depends on integrated control among development, environment, and chemical expression.

### 2.7. Current Limitations and Future Directions

An important limitation in studies of chemical ontogeny is the absence of standardized developmental descriptors across taxa, organs, tissues, and experimental systems. Many phytochemical and metabolomic studies describe the sampled material only as young, mature, adult, flowering, or fruiting, without defining the developmental criteria used to assign these categories. This imprecision restricts comparisons among studies and may obscure biologically relevant differences among whole-plant developmental stages, organ-specific maturation, tissue differentiation, and phenological events [56,57,260,261,262]. Future studies should combine species-specific descriptors with broader ontological systems, such as vocabularies of plant developmental stages and standardized growth scales, in order to improve semantic consistency and comparability among studies. The Plant Ontology provides controlled terms for plant structures and developmental stages, whereas MIAPPE was developed to harmonize metadata in plant phenotyping experiments [260,261,263,264].

Reproducibility remains limited by incomplete metadata on genotype, accession, cultivation conditions, plant age, organ position, developmental stage, harvest time, postharvest handling, extraction method, analytical platform, and data-processing workflow. In metabolomics, such omissions are particularly problematic because small differences in sampling, storage, extraction, ionization, chromatographic conditions, and *feature* annotation may profoundly alter the apparent chemical profile [265,266,267,268]. Minimum reporting standards for metabolomics emphasize the need to document sample preparation, analytical procedures, quality control, metabolite identification, and data processing. The *FAIR* principles further reinforce that datasets and workflows should be findable, accessible, interoperable, and reusable, including by computational systems [264,266,267,269].

Given these limitations, Table 1 consolidates operational recommendations for ontogeny-aware phytochemical research, covering study delimitation, botanical identity, sampling design, definition of phase and stage, organography, temporal resolution, ecophysiological metadata, morphoanatomical integration, cultivation conditions, postharvest handling, analytical platform, marker validation, chemometrics, and ecofunctional interpretation. These recommendations seek to reduce the risk of confusing ontogenetic variation with seasonality, environment, genotype, organ-specific maturation, or analytical artifacts, while aligning plant material description with the minimum reporting standards in metabolomics and with the principles of data reproducibility and interoperability [2,4,20,56,57,265,266,269].

Chemical ontogeny is rarely isolated from environmental and genetic sources of variation. Seasonality, photoperiod, temperature, water availability, soil chemistry, herbivory, pathogen pressure, plant density, and genotype may interact with developmental stage, producing chemical patterns that may be mistakenly interpreted as purely ontogenetic [2,15,16,51]. This is particularly relevant in the case of medicinal plants, used in the treatment of diseases and in healthcare, and aromatic plants, species characterized by the emission of volatile organic compounds [4]. In these types of plants, the same metabolite may respond simultaneously to age, tissue differentiation, phenological phase, abiotic stress, and genetic context [4,20,270,271]. Therefore, future studies should adopt factorial designs, common-garden experiments, clonal materials, controlled-environment cultivation, repeated sampling, and statistical models capable of partitioning developmental, environmental, seasonal, and genotypic effects [15,16,183].

The literature is dominated by studies based on cross-sectional sampling, comparing different individuals or organs collected at discrete developmental moments. Although useful, this approach only offers a fragmented view of metabolic trajectories. Chemical ontogeny requires longitudinal and time-series designs capable of following, over time, the same genotype, population, organ cohort, or developmental sequence [2,272,273]. Such designs would allow for the identification of transient metabolites, biosynthetic intermediates, metabolic inflection points, developmental windows of maximum bioactivity, and stage-specific chemical markers [4,19,39]. Temporally resolved metabolomics, combined with transcriptomics, proteomics, anatomical characterization, and bioassays, should become a priority for understanding causality, rather than merely describing chemical variation [271,273,274,275].

The field also requires databases capable of integrating chemical profiles with developmental descriptors, voucher information, georeferenced collection data, environmental metadata, taxonomic identity, organ and tissue ontologies, analytical parameters, and bioactivity results. The repositories and workflows currently available in metabolomics are valuable but remain insufficiently adapted to represent ontogenetic trajectories in plants [276,277,278]. Future databases should support temporally indexed samples, developmental-stage annotations, controlled vocabularies, deposition of raw data, spectral metadata, molecular networking results, and interoperable connections among metabolomic, genomic, transcriptomic, phenomic, and pharmacognostic datasets [215,216,264,269]. This would reduce redundancies, improve reproducibility, and enable meta-analyses across species, organs, environments, and chemical classes [268,269,277,278].

The next phase of research on chemical ontogeny will likely involve predictive and artificial-intelligence-assisted frameworks. Machine learning, explainable artificial intelligence, network analysis, and multi-omics integration may help model developmental trajectories, predict optimal harvest windows, identify robust quality markers, prioritize bioactive compounds, and distinguish ontogenetic variation from environmental noise [208,271,278,279]. Recent reviews indicate that AI is increasingly relevant to metabolomics, phytochemical research, structural elucidation, metabolic profiling, multi-omics integration, and natural product discovery [279,280,281]. However, these approaches will be reliable only if they are trained on standardized, well-annotated, chemically validated, and developmentally resolved datasets, reinforcing the need for harmonized descriptors, *FAIR* data architectures, interoperable repositories, and transparent computational workflows [264,269,278,279].

## 3. Materials and Methods

This review was conducted from the perspective of an *integrative review*, with a *narrative approach* and *thematic synthesis*, aiming to gather, articulate, and discuss contemporary knowledge on *chemical ontogeny* in plants. This design was adopted because it allows for the critical integration of conceptual, experimental, methodological, and applied studies, especially in fields in which the available data are heterogeneous with regard to plant models, developmental stages, metabolite classes, analytical methods, and ecological or pharmacognostic contexts investigated [282,283,284,285,286,287].

The central focus of the review was to understand how plant developmental phases/stages influence the dynamics of *specialized metabolism* and how these changes affect ecological interactions, the interpretation of phytochemical data, quality control of botanical raw materials, *chemophenetics*, *chemotaxonomy*, and bioprospecting strategies. The adopted approach considers that specialized metabolites should not be interpreted merely as products of specialized metabolism, but as dynamic components of metabolic networks associated with defense, regulation, development, ecological adaptation, and tissue-specific differentiation [9,10,11,15,16].

The construction of the bibliographic corpus was broad, critical, and interpretative in nature. The Scopus, PubMed, and Google Scholar databases were consulted, prioritizing publications in English made available between January 2021 and March 2026, in order to encompass the most recent scientific production on specialized metabolism, plant metabolomics, integrated omics, chemical ecology, and the quality control of botanical products (Table S1). Studies published before this time frame were selectively incorporated when they presented conceptual, historical, methodological, or mechanistic relevance to the foundation of the discussion, especially regarding the ontogeny of plant defense and the theoretical organization of specialized metabolism.

The search strategy was guided by combinations of descriptors related to ontogeny, plant development, and specialized metabolism; Boolean combinations among these descriptors were used. The selection of studies did not follow a strictly systematic protocol, with prior registration, meta-analysis, or formal risk-of-bias assessment. However, explicit criteria of thematic relevance, conceptual relevance, methodological contribution, and interpretative applicability were adopted. Priority was given to studies addressing qualitative or quantitative changes in specialized metabolites throughout plant development, including variations among juvenile, vegetative, reproductive, senescent, and immediate post-developmental stages, as well as investigations on organ-specific, tissue-specific, or cell-specific differences in the production, accumulation, transport, or transformation of these metabolites (Table S1).

After reading the titles, abstracts, and, when necessary, full texts, the selected studies were organized according to their contribution to the objectives of the review. Based on the generated extraction matrix, the selected recent studies were systematized in Table S2, which presents representative examples of ontogenetic variation in specialized metabolites across different botanical families, organs, developmental phases or stages, chemical classes, and analytical platforms. This systematization shows that chemical ontogeny is not restricted to a specific taxonomic group, organ, or metabolic class, but involves recurrent changes in flavonoids, terpenoids, alkaloids, saponins, carotenoids, anthocyanins, and other specialized metabolites throughout the plant life cycle [4,15,19,21].

Detailed data search and extraction procedures, additional selection information, and other methodological details can be accessed in the Supplementary Materials. Finally, ChatGPT-5 (OpenAI, San Francisco, CA, USA) was used as an auxiliary tool during manuscript preparation, exclusively to support textual correction, translation of excerpts, improvement of linguistic clarity, and preliminary organization of ideas. All scientific information, interpretations, methodological decisions, bibliographic selection, and conceptual formulations were critically evaluated, revised, and validated by the authors, who assume full responsibility for the final content of the work.

## 4. Conclusions

In lyrical tones, in the life of a plant, chemistry is not a silent inventory of molecules, but a temporal language through which development, the environment, and ecological function are continuously inscribed. From the earliest juvenile tissues to reproductive organs, ripening fruits, seeds, and senescent structures, the plant does not merely grow; it re-organizes its own chemical existence.

Technically speaking, this review emphasizes that plant chemistry is not a static inventory of molecules but a temporally structured manifestation of development. From juvenile tissues to senescent structures, plants do not merely grow; they continuously reorganize their chemical composition. Chemical ontogeny therefore provides an integrative framework for understanding specialized metabolism as a dynamic component of plant development. Developmental transitions reshape biosynthetic capacity, metabolite allocation, tissue-specific compartmentalization, ecological functions, and pharmacognostic relevance.

This perspective has direct implications for phytochemistry, pharmacognosy, chemical ecology, quality control, chemophenetics, and bioprospecting. Developmental stage can influence chemical markers, bioactivity, authenticity, safety, and reproducibility by modifying metabolite abundance, composition, and stability. Therefore, harvest timing, marker selection, bioassay interpretation, and botanical standardization should explicitly incorporate developmental stage as a methodological variable.

Future studies should prioritize developmentally resolved sampling, standardized stage descriptors, appropriate experimental controls, and robust analytical validation to distinguish ontogenetic variation from seasonal, environmental, and genotypic effects. Targeted analyses, metabolomics, chemical imaging, transcriptomics, multi-omics integration, and computational approaches may be selected according to the research question and available infrastructure. By incorporating developmental context into phytochemical research, chemical ontogeny can improve reproducibility, botanical quality assessment, and the identification of ecologically, pharmacologically, and biotechnologically relevant metabolic patterns.

## Figures and Tables

**Table 1:** Operational recommendations for designing, reporting, and interpreting ontogeny-aware phytochemical studies.

Domain	Best-Practice Recommendation	Minimum Operational Standard	Preferred/Advanced Standard	Required Metadata/Data to Report	Analytical/Statistical Considerations	Risk If Ignored	Reporting Checklist Item
Study delimitation	State whether the study tests ontogenetic effects, phenological effects, environmental effects, or their interaction.	Define the primary developmental contrast before sampling and specify the target metabolite class or metabolomic question.	Use a causal diagram or pre-specified analytical plan separating plant age, plant stage, organ stadium, season, and environment.	Research question; hypothesis; target organs; metabolite classes; intended application; expected confounders.	Model stage/stadium as explicit factors rather than post hoc descriptors. Include interaction terms when environment or season is not controlled.	Ontogenetic differences may be confused with seasonality or cultivation effects.	Objective explicitly identifies the developmental scale under analysis.
Botanical identity and traceability	Guarantee taxonomic authenticity and traceability of all plant material.	Report accepted name, authority, family, voucher number, herbarium, collector, and collection/cultivation origin.	Add genotype, chemotype, accession, clonal lineage, population history, DNA barcode, and photographic voucher when relevant.	Species; authority; voucher; herbarium; accession/genotype; source population; cultivation history.	Treat genotype/population as random or fixed effects when multiple origins are included.	Chemical variation may be incorrectly attributed to ontogeny when it reflects taxonomic or genetic heterogeneity.	Voucher and biological source are fully traceable.
Ontogenetic sampling design	Sample along a developmentally meaningful sequence rather than using a single poorly described collection moment.	Include at least three developmental time points or biologically justified stages/stadia when the goal is trajectory inference.	Use repeated sampling, common-garden or controlled-environment designs, and balanced factorial combinations of stage × organ × environment.	Plant age; developmental stage; organ stadium; phenological condition; sampling interval; developmental scale used.	Use mixed models, PERMANOVA, trajectory analysis, or multivariate regression with plant individual/block as random factors.	Transient compounds, biosynthetic intermediates, and stage-specific signatures may remain undetected.	Sampling captures the relevant developmental window.
Replication and experimental unit	Define the true biological replicate and avoid treating subsamples or injections as independent plants.	Use independent plants as biological replicates; document technical replicates separately.	Use blocked designs across plots, benches, batches, populations, or seasons; include power justification when feasible.	Plant ID; plot/block; biological replicate number; subsampling scheme; technical replicate number; batch ID.	Avoid pseudoreplication. Use nested or hierarchical models when organs are sampled within plants.	Inflated degrees of freedom and false marker-stage associations.	Biological, technical, and analytical replication are distinguished.
Definition of stage and stadium	Distinguish plant developmental stage from organ or tissue developmental stadium.	Define stage at the whole-plant level and stadium at the organ/tissue level using observable criteria.	Adopt a species-specific or BBCH-like scale, supported by morphometric, anatomical, and physiological descriptors.	Stage code; stadium code; criteria; organ position; tissue age; photographs; anatomical confirmation when used.	Analyze stage and organ stadium as separate factors when both vary in the same plant.	A reproductive plant may contain young, mature, and senescent organs with distinct metabolic profiles.	Terminology separates whole-plant stage from organ/tissue stadium.
Sampling position and organography	Control the position of the organ within plant architecture.	Report organ position, node number, branch order, leaf rank, inflorescence position, or root segment.	Use architectural mapping, standardized organographic diagrams, or image-based phenotyping.	Node/rank; branch order; canopy position; exposure; distance from meristem; organ age; developmental sequence.	Include organ position as a covariate or nested factor when multiple positions are sampled.	Positional gradients may be misread as ontogenetic effects.	Sampling position is reproducible and anatomically explicit.
Temporal resolution	Use sampling intervals compatible with the expected speed of developmental and metabolic change.	Record date, time of day, season, and developmental duration since germination, bud break, anthesis, or fruit set.	Use chronobiological control, circadian sampling windows, and repeated measures for fast-changing metabolites.	Calendar date; time; plant age; organ age; phenological event anchor; season; photoperiod.	Account for circadian and seasonal effects; avoid comparing morning and afternoon collections as developmental contrasts.	Diurnal or seasonal variation may mask or mimic ontogenetic differences.	Time metadata are sufficient to reproduce the collection window.
Ecophysiological metadata	Measure physiological state at or near the time of sampling.	At minimum, record visible stress, water status proxies, temperature, light conditions, and cultivation/field context.	Measure photosynthesis, stomatal conductance, chlorophyll fluorescence, SPAD, water potential, and relative water content.	Fv/Fm; SPAD; gas exchange; leaf water potential; RWC; stress symptoms; microclimate.	Use physiological variables as covariates or explanatory layers in multivariate models.	Chemical differences may be attributed to development while reflecting stress physiology.	Physiological condition is measured or explicitly documented.
Morpho-anatomical integration	Link chemical profiles to visible and microscopic differentiation of organs and tissues.	Record morphometric traits and developmental photographs for each sampled stadium.	Include microscopy, secretory-structure quantification, trichome density, tissue thickness, vascularization, or histochemistry.	Leaf area; thickness; trichome density; secretory cavities; laticifers; idioblasts; microscopy method; image IDs.	Use correlation, multiblock analysis, or integrative ordination to relate anatomy and chemistry.	Metabolic shifts may be interpreted without considering emergence or maturation of biosynthetic structures.	Morphological/anatomical criteria support stadium assignment.
Agricultural and cultivation metadata	Document agronomic factors that may interact with ontogeny.	Report cultivation system, substrate/soil, irrigation, fertilization, pest management, plant density, and harvest method.	Use controlled agronomic protocols or factorial designs testing stage × cultivation factor interactions.	Soil/substrate; nutrients; irrigation; light/shading; greenhouse/field; pesticide/biostimulant use; plant density.	Model agricultural factors or hold them constant across stages/stadia.	Management effects may be confused with developmental chemistry.	Cultivation context is sufficient for reproducibility and scaling.
Sample handling and post-harvest control	Prevent post-harvest transformation from distorting ontogenetic signatures.	Standardize harvest-to-quench time, drying, storage, grinding, moisture determination, and extraction mass.	Use cryogenic quenching or validated drying protocols; monitor stability under processing and storage conditions.	Fresh/dry mass; moisture; drying temperature/time; storage temperature; storage duration; particle size; extraction delay.	Evaluate processing as a batch factor; include QC samples to detect drift after extraction or storage.	Observed differences may result from degradation, enzymatic conversion, volatilization, or oxidation.	Post-harvest chain is standardized and reported.
Analytical workflow	Select analytical platforms consistent with the chemical class and developmental question.	Report extraction method, instrument platform, chromatographic conditions, ionization/detection mode, and data preprocessing.	Combine targeted quantification with untargeted profiling, molecular networking, dereplication, and orthogonal confirmation when needed.	Platform; method; solvent; internal standards; QC design; blanks; batch order; preprocessing parameters; annotation level.	Randomize injections; use pooled QC; monitor retention time, mass accuracy, response drift, and carryover.	Non-biological analytical drift may be mistaken for developmental structure.	Analytical quality assurance is transparent.
Marker validation	Validate markers across developmental windows rather than assuming stability from a single stage.	Confirm detection, repeatability, and quantitative performance for each proposed marker in all relevant stages/stadia.	Assess stage specificity, linearity, LOD/LOQ, precision, recovery, stability, matrix effects, and inter-batch/instrument transferability.	Marker identity; annotation confidence; calibration; validation metrics; stage distribution; biological relevance.	Use ROC curves, effect sizes, mixed models, PCA loading stability, or external validation sets when markers are diagnostic.	A marker may fail in juvenile, senescent, or reproductive material, compromising authenticity and quality control.	Marker suitability is demonstrated for the intended developmental material.
Chemometrics and data integration	Use statistical workflows capable of separating ontogenetic structure from confounding sources of variance.	Report preprocessing, normalization, transformation, missing-value rules, scaling, and model validation.	Use validated multivariate trajectories, ASCA, network analysis, or multiblock/multi-omics models only when justified by the question and supported by adequate sample size; single-platform models remain appropriate.	Data matrix; metadata matrix; preprocessing; model parameters; validation strategy; code/software.	Evaluate explained variance, classification accuracy, permutation tests, cross-validation, and model stability.	Overfitted models may produce non-reproducible developmental markers.	Statistical workflow is reproducible and linked to metadata.
Ecofunctional and translational interpretation	Interpret ontogenetic chemistry in relation to function, quality, or use, not only as compositional variation.	Discuss how each stage/stadium affects ecological interactions, pharmacognostic quality, safety, yield, or bioprospecting value.	Link chemical changes to bioassays, ecological assays, gene expression, anatomical evidence, or quality specifications.	Intended use; biological activity; quality specification; target markers; harvest recommendation; limitations.	Test whether chemical differences correspond to functional differences; avoid extrapolating bioactivity from chemistry alone.	Harvest recommendations may be scientifically unsupported or operationally irreproducible.	Interpretation connects chemistry, development, and application.

## Data Availability

No new primary data were generated in this study. All data analyzed in this integrative review were obtained from the published sources cited in the manuscript. The data supporting the findings are provided within the article and its Supplementary Materials (Tables S1 and S2).

## Supplementary Materials

The following supporting information can be downloaded at: https://www.mdpi.com/article/10.3390/plants15182756/s1, Table S1. PRISMA-2020 pathway and corpus construction, starting from 2568 database records and reaching the final analytical corpus; Table S2. Representative examples of ontogenetic changes in specialized metabolites (2021–2026); Figure S1. Interpretive dimensions of Figure 2; Figure S2. Chemical Trajectories Across Reproductive Development in *Piper mollicomum*. Empirical example of chemical ontogeny [60].

## Abbreviations

The following abbreviations are used in this manuscript:

ABC	ATP-binding cassette
AI	Artificial intelligence
APC	Article processing charge
ASCA	ANOVA-simultaneous component analysis
BBCH	Biologische Bundesanstalt, Bundessortenamt and Chemical Industry phenological scale
bHLH	Basic helix–loop–helix
CAPES	Coordenação de Aperfeiçoamento de Pessoal de Nível Superior
CC BY	Creative Commons Attribution
COI1	CORONATINE INSENSITIVE 1
CT-Agro	Fundo Setorial de Agronegócio
DAD	Diode-array detection
DESI-MSI	Desorption electrospray ionization mass spectrometry imaging
DFR	Dihydroflavonol 4-reductase
DIMBOA	2,4-Dihydroxy-7-methoxy-1,4-benzoxazin-3-one
DIMBOA-Glc	DIMBOA glucoside
DIM2BOA-Glc	2,4-Dihydroxy-7,8-dimethoxy-1,4-benzoxazin-3-one glucoside
DNA	Deoxyribonucleic acid
ERF	Ethylene response factor
FAIR	Findable, accessible, interoperable, and reusable
FBMN	Feature-based molecular networking
FINEP	Financiadora de Estudos e Projetos
FNDCT	Fundo Nacional de Desenvolvimento Científico e Tecnológico
FT-ICR-MS	Fourier transform ion cyclotron resonance mass spectrometry
FTIR	Fourier transform infrared spectroscopy
GC-FID	Gas chromatography with flame ionization detection
GC-MS	Gas chromatography-mass spectrometry
GC-MS/MS	Gas chromatography-tandem mass spectrometry
GNPS	Global Natural Products Social Molecular Networking
HCA	Hierarchical cluster analysis
HPLC	High-performance liquid chromatography
HPLC-DAD	High-performance liquid chromatography with diode-array detection
HPLC-HRESIMS	High-performance liquid chromatography-high-resolution electrospray ionization mass spectrometry
HPLC-MS	High-performance liquid chromatography-mass spectrometry
HPTLC	High-performance thin-layer chromatography
HS-SPME	Headspace solid-phase microextraction
HS-SPME/GC-MS	Headspace solid-phase microextraction coupled to gas chromatography-mass spectrometry
ID	Identifier
IMS-MS	Ion mobility spectrometry-mass spectrometry
JA	Jasmonic acid
JA-Ile	Jasmonoyl-isoleucine
JAZ	JASMONATE ZIM-domain protein
LC-HRMS	Liquid chromatography-high-resolution mass spectrometry
LC-MS	Liquid chromatography-mass spectrometry
LC-MS/MS	Liquid chromatography-tandem mass spectrometry
LOD	Limit of detection
LOESS	Locally estimated scatterplot smoothing
LOQ	Limit of quantification
MALDI	Matrix-assisted laser desorption/ionization
MALDI-MSI	Matrix-assisted laser desorption/ionization mass spectrometry imaging
MCTI	Ministério da Ciência, Tecnologia e Inovação
MIAPPE	Minimum Information About a Plant Phenotyping Experiment
miR156	microRNA156
miR156b	microRNA156b
MS	Mass spectrometry
MS/MS	Tandem mass spectrometry
MS2LDA	MS2 Latent Dirichlet Allocation
MSI	Mass spectrometry imaging
MYB	MYB transcription factor family
MYC2	MYC2 transcription factor
NAC	NAM, ATAF1/2, and CUC2 transcription factor family
NIC2	Nicotine biosynthesis 2 locus
NMR	Nuclear magnetic resonance
^1^H NMR	Proton nuclear magnetic resonance
NPClassifier	Natural Products Classifier
OPLS-DA	Orthogonal partial least squares discriminant analysis
PCA	Principal component analysis
PERMANOVA	Permutational multivariate analysis of variance
PLS	Partial least squares
PLS-DA	Partial least squares discriminant analysis
PRPPG	Pró-Reitoria de Pesquisa e Pós-Graduação
QC	Quality control
QqQ	Triple quadrupole
RNA	Ribonucleic acid
RNA-Seq	RNA sequencing
ROC	Receiver operating characteristic
RWC	Relative water content
SPAD	Soil plant analysis development chlorophyll index
SPL	SQUAMOSA promoter-binding protein-like
SPL9	SQUAMOSA promoter-binding protein-like 9
UHPLC	Ultra-high-performance liquid chromatography
UHPLC-DAD	Ultra-high-performance liquid chromatography with diode-array detection
UHPLC-HRMS/MS	Ultra-high-performance liquid chromatography-high-resolution tandem mass spectrometry
UHPLC-QqQ-MS/MS	Ultra-high-performance liquid chromatography-triple-quadrupole tandem mass spectrometry
UPLC	Ultra-performance liquid chromatography
UPLC-MS/MS	Ultra-performance liquid chromatography-tandem mass spectrometry
UV	Ultraviolet
WRKY	WRKY transcription factor family
