# Supplementary material for: Chemical Ontogeny in Plants: Developmental Dynamics of Specialized Metabolism and Implications for Ecology, Quality Control, and Bioprospecting
Source: Plants (Basel). 2026 Sep 9;15(18):2756. doi: 10.3390/plants15182756 (PMC13610671; doi:10.3390/plants15182756)

Table S1. PRISMA-ScR pathway and corpus construction, starting from 2,568 database records and reaching the final analytical corpus.

PRISMA or operational step	Value
Records identified through databases	2,568
Duplicates removed	8
Records remaining after duplicate removal	2,560
Records excluded during preliminary operational refinement	2,313
Records screened by title/abstract	247
Records excluded after title/abstract screening	186
Records referred to operational full-text eligibility assessment	61
Reports assessed in the operational full-text stage	61
Reports excluded in the operational full-text stage	7
Database records included after operational full-text assessment	54
Qualified manual/theoretical records	132
Total analytical records included in the review	186

Figure S1. Interpretive dimensions of figure 2

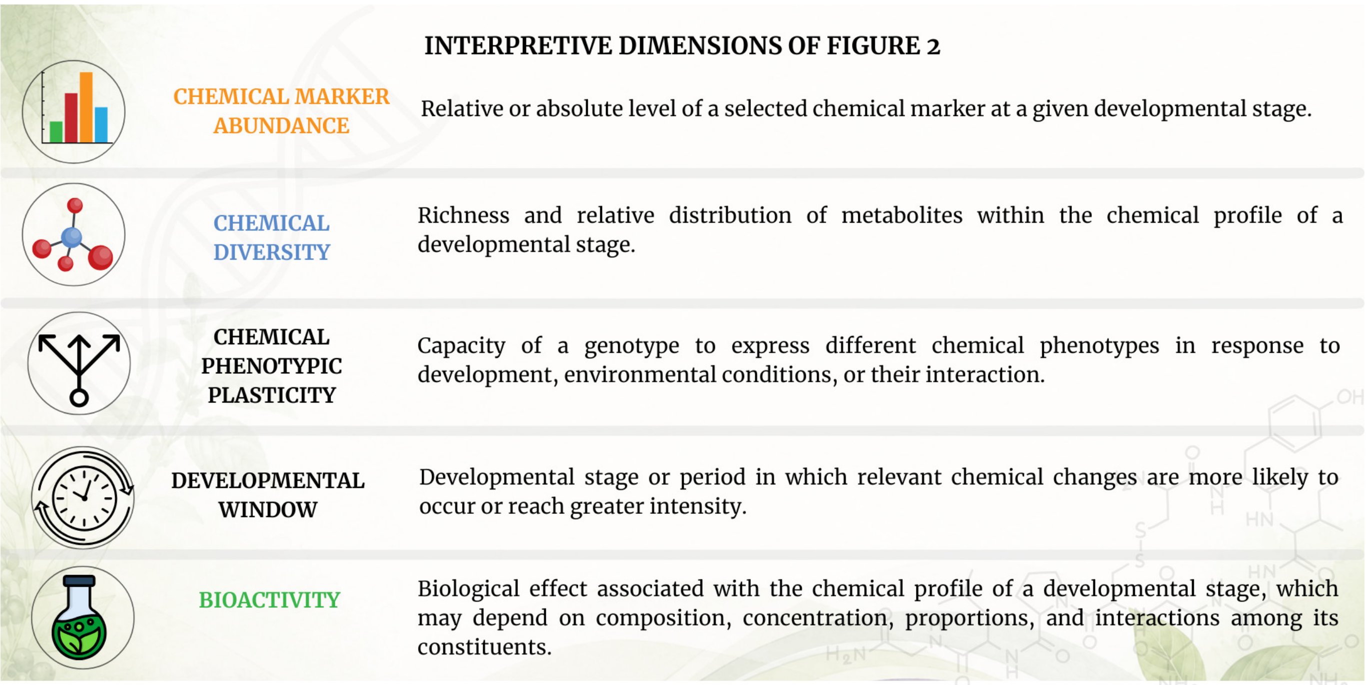

Figure S2. Chemical Trajectories Across Reproductive Development in *Piper mollicomum*. ***Empirical example of chemical ontogeny (de Brito-Machado et al., 2022)***

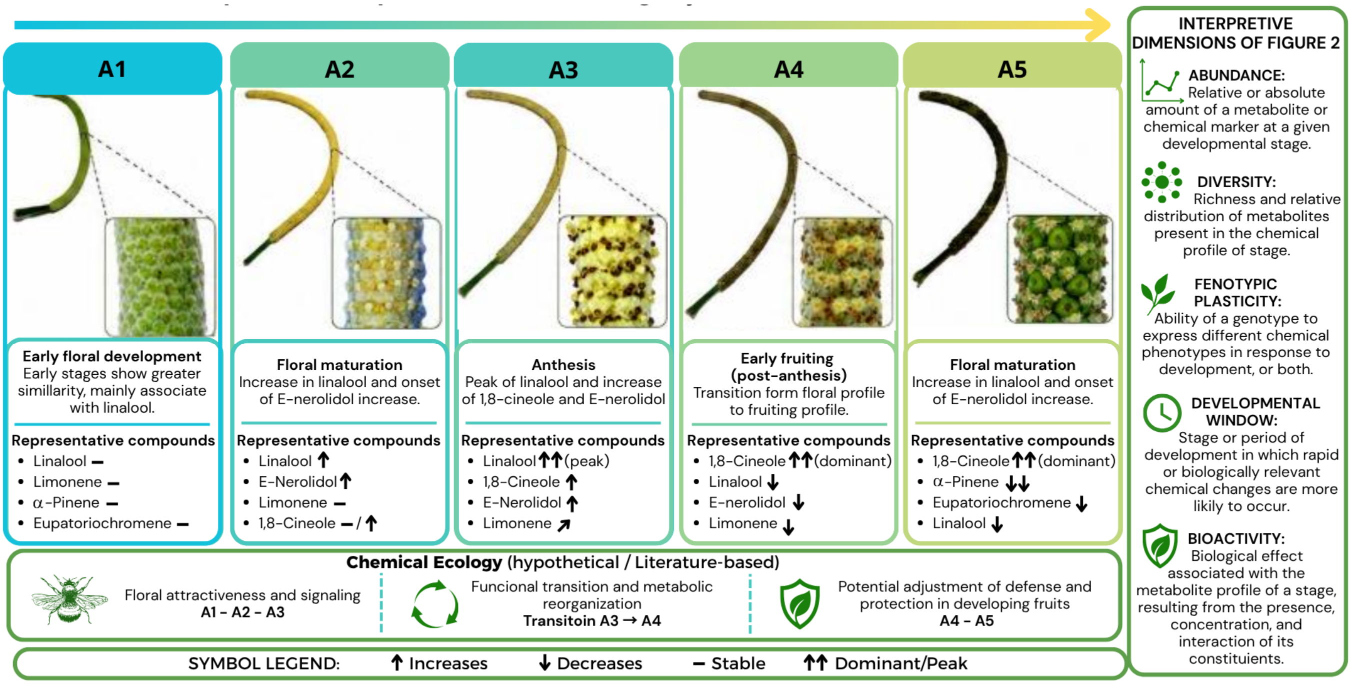

Supplement: Supplementary file 1 [file plants-15-02756-s001.zip › plants-4427692-Supplementary.pdf]
